# Supplementary material for: Time-resolved interactome profiling deconvolutes secretory protein quality control dynamics
Source: Mol Syst Biol. 2024 Aug 5;20(9):1049–75. doi: 10.1038/s44320-024-00058-1 (PMC11369088; doi:10.1038/s44320-024-00058-1)
Supplement: Supplementary file 1 — Appendix [file 44320_2024_58_MOESM1_ESM.pdf]

Appendix

Time-Resolved Interactome Profiling Deconvolutes Secretory Protein Quality Control Dynamics

Madison T. Wright, Bibek Timalsina, Valeria Garcia Lopez, Jake Hermanson, Sarah Garcia, Lars Plate*
*For correspondence: lars.plate@vanderbilt.edu

Table of Content

Appendix Figure S1 - Validation of FRT stable cell lines 2
Appendix Figure S2 - Two-stage enrichment strategy requires FLAG-tag Tg and pulse-labeling with Hpg 3
Appendix Figure S3 – (-) Biotin pulldown carrier samples allow for identification of Tg interactors 5
Appendix Figure S4 – Summary of Tg TRIP Data –scaled heatmap. 6
Appendix Figure S5 - Validation of Tg-NLuc stable cell lines 9
Appendix Figure S6 - Summary of siRNA screening data 11
Appendix Figure S7 - Mutant Tg shows selective enrichment with TEX264 in a screen of multiple ER-phagy receptors 13
Appendix Figure S8 - TRIP of C1264R Tg-FT FRT cells with pharmacological VCP inhibition 14

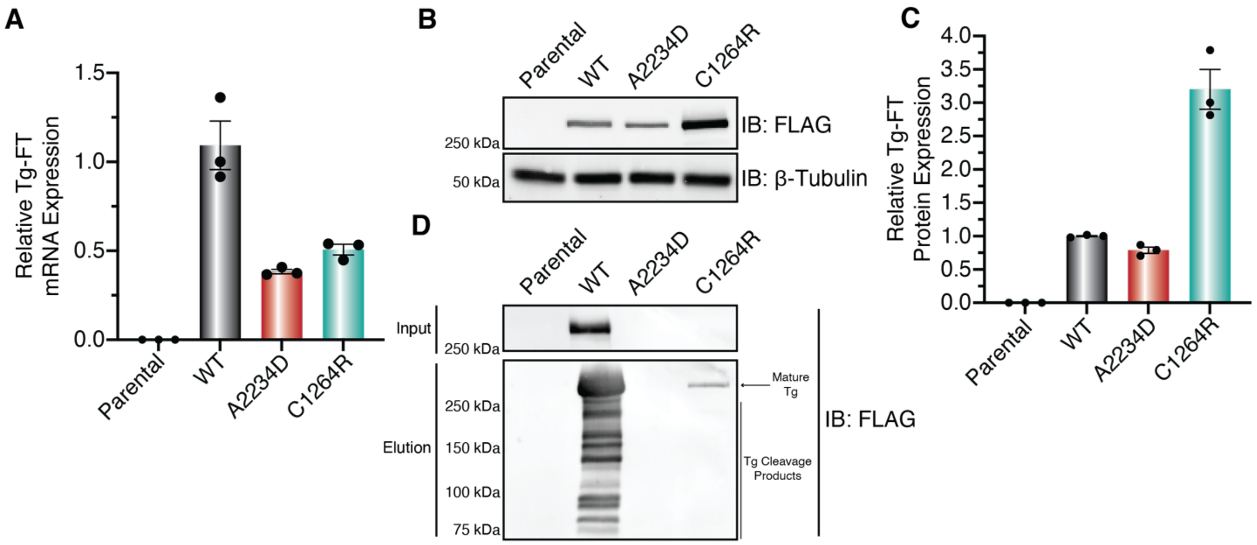

Appendix Figure S1 - Validation of FRT stable cell lines

(A) Relative expression of Tg-FT RNA from engineered isogenic FRT cells measured by qRT-PCR. After transfections with Tg-FT pcDNA and flp recombinase pOG44, cells were placed under selection with Hygromycin B (100 µg/mL) to select site-specific recombinants. Resistant clonal lines were sorted into single cell colonies using flow cytometry and screen for Tg-FT expression. Data was first normalized to a GAPDH loading control followed by normalization to median WT Tg-FT expression and represented as mean ± SEM. Primers for detection described in **Table EV2**.

(B) Western blot analysis of Tg-FT expression in lysates from engineered isogenic FRT cells. FT signal detected via M2-FLAG antibody. FT is only detectable in isogenic cells co-transfected with Tg-FT pcDNA and flp recombinase pOG44, while FT signal is absent in parental cells. β-Tubulin used as a loading control.

(C) Quantification of relative Tg-FT expression in engineered FRT cells measured by Western blot analysis in (B). Data was first normalized to the β-Tubulin used as a loading control, followed by normalization to median WT Tg-FT expression and represented as mean ± SEM.

(D) Western blot analysis of Tg-FT expression in media from engineered FRT cells. WT Tg-FT is efficiently secreted and detectable in both media inputs and after immunoprecipitation. C1264R secretion is drastically decreased compared to WT Tg-FT and is only detectable after immunoprecipitation, while A2234D secretion is not detectable in inputs or after immunoprecipitation. Mature Tg and Tg cleavage products are annotated in elution samples.

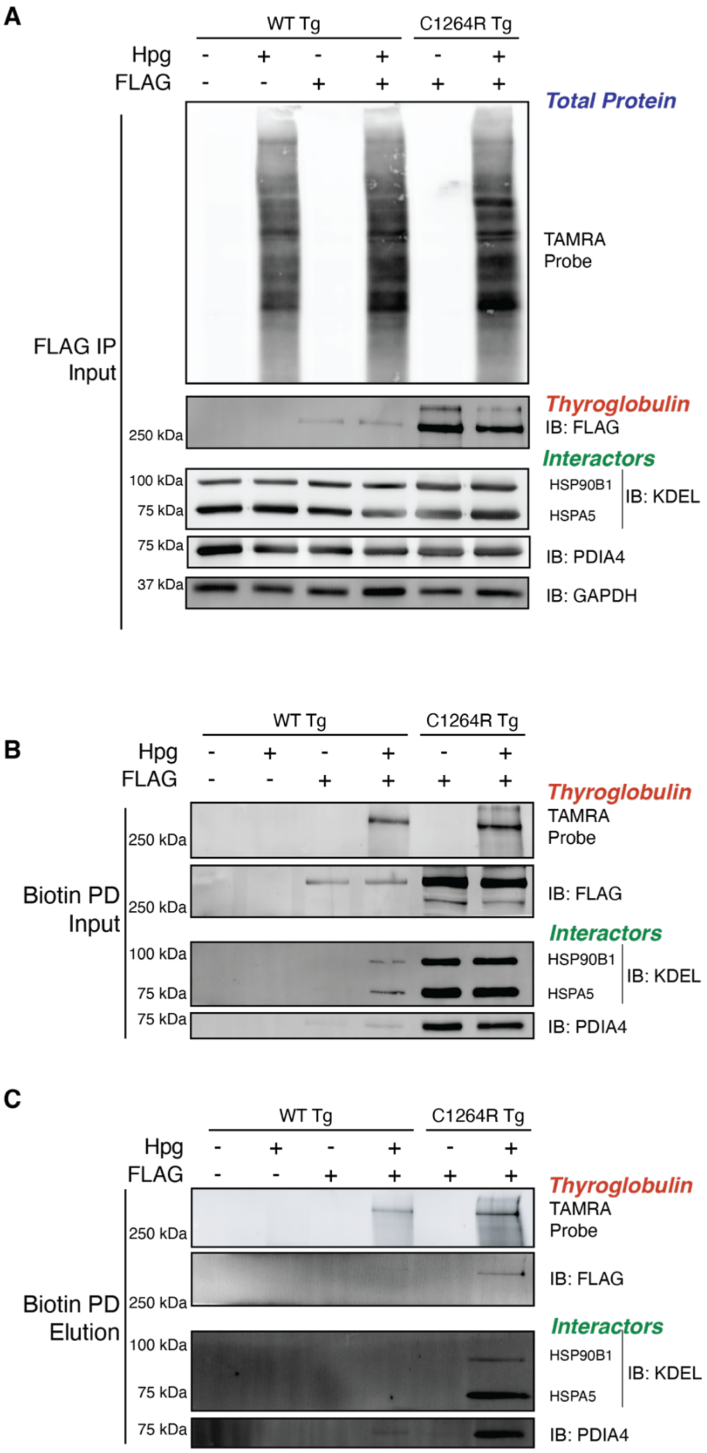

Appendix Figure S2 - Two-stage enrichment strategy requires FLAG-tag Tg and pulse-labeling with Hpg

(A-C) Western blot analysis of Tg purification after 4 h of continuous Hpg labeling and functionalization with TAMRA-Azide-PEG-Desthiobiotin probe. All conditions were crosslinked with DSP (0.5mM) for 10 minutes to capture transient interactions. (A) FLAG IP inputs showing TAMRA labeled proteins and immunoblots of Tg (IB: FLAG), interactors, and loading control (IB:

GAPDH). (B) FLAG IP elutions showing TAMRA labeled proteins and immunoblots of Tg and interactors. Samples subsequently underwent biotin pulldown. (C) Biotin pulldown elutions with TAMRA labeled proteins, Tg, and interactors.

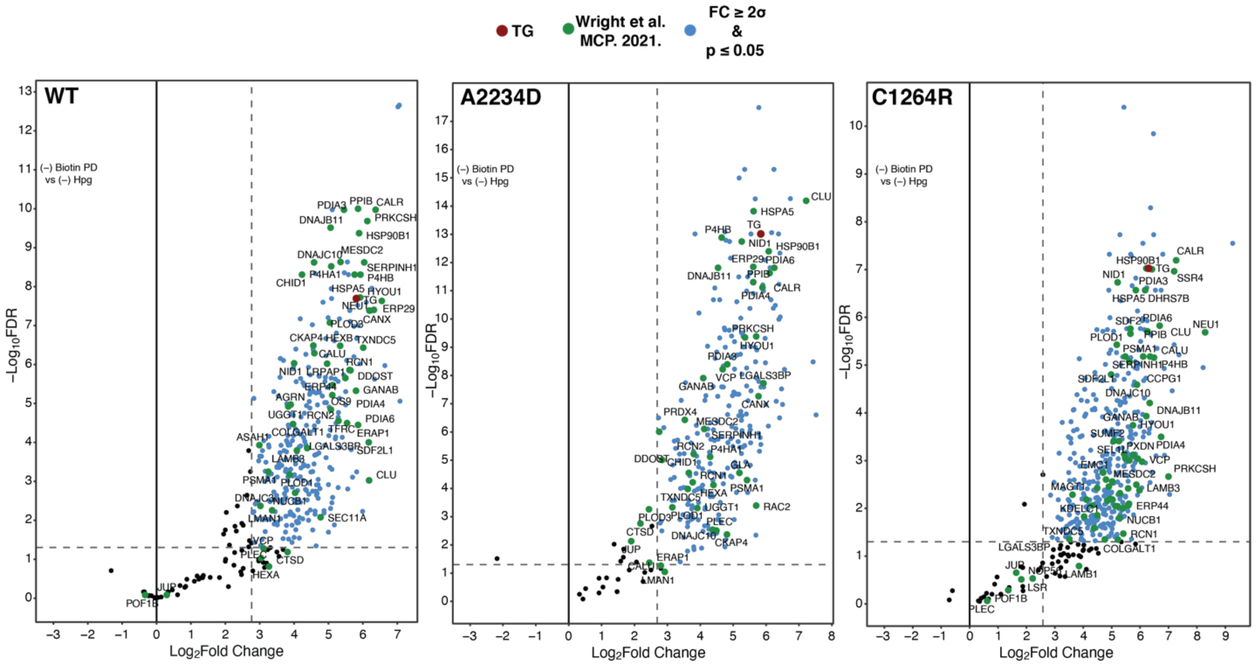

Appendix Figure S3 – (-) Biotin pulldown carrier samples allow for identification of Tg interactors

Volcano plots for the comparison of (-) biotin pulldown vs (-) Hpg samples to identify Tg interactors in FRT cells. Plots show the average log2 difference vs FDR estimation (Benjamini-Hochberg). Data was processed through the DEP pipeline (script available at github.com/wrighmt1/2022_TRIP) (Zhang et al., 2018). (-) biotin pulldown samples were pulse-labeled with Hpg (200µM) for 1 hour and cultured in normal F12 media throughout the remainder of the 3-hour chase period. Cells were cross linked with DSP (0.5mM) for 10 minutes to capture transient proteoastasis network interactions. Lysates were functionalized with TAMRA-Azide-PEG-Desthiobiotin probe using CuAAC Click reaction. (-) biotin pulldown samples then underwent immunoprecipitation and were processed for mass spectrometry. (-) Hpg samples were processed through the entire dual affinity purification TRIP workflow including 3 hour chase period, absent Hpg labeling, and used for enrichment analysis. Enriched proteins were determined based on those with a log2 fold change of 2σ and Benjamini-Hochberg adjusted p-value (false discovery rate) of 0.05. Dashed lines indicate cutoffs for log2 fold change and adjusted p-values. Proteins annotated in blue are above log2 fold change and adjusted p-value cutoffs and considered Tg interactors. Proteins annotated in green were identified as Tg interactors in our previous mass spectrometry dataset (Wright et al., 2021). Tg is annotated in red. Previously identified Tg interactors are heavily enriched in the dataset along with several novel interactors. Full MS data can be found **Dataset EV1** and DEP output can be found in **Dataset EV2**.

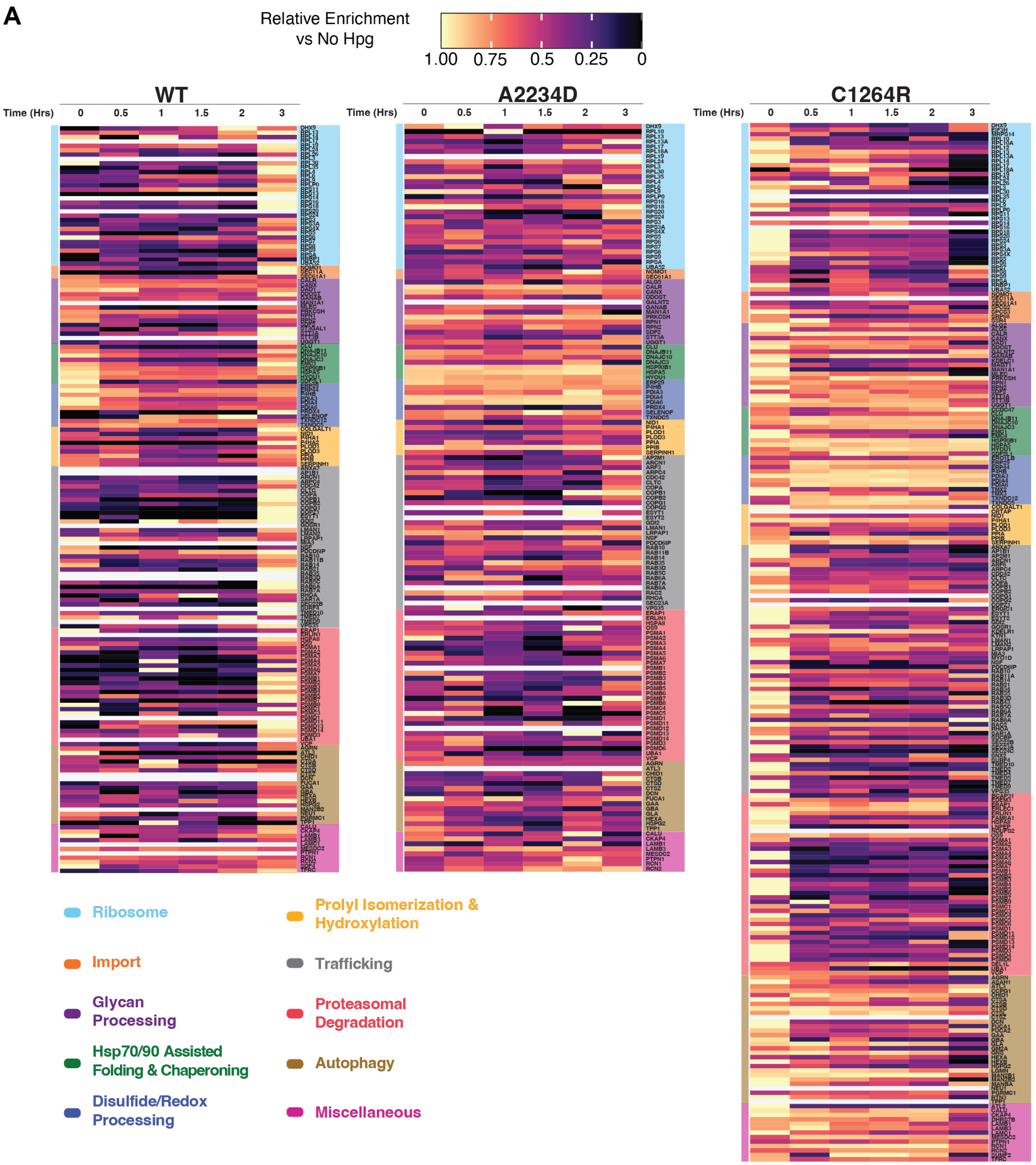
A
Relative Enrichment
vs No Hpg
1.00 0.75 0.5 0.25 0
WT
A2234D
C1264R
Time (Hrs)
0 0.5 1 1.5 2 3
Ribosome
Import
Glycan Processing
Hsp70/90 Assisted Folding & Chaperoning
Disulfide/Redox Processing
Prolyl Isomerization & Hydroxylation
Trafficking
Proteasomal Degradation
Autophagy
Miscellaneous

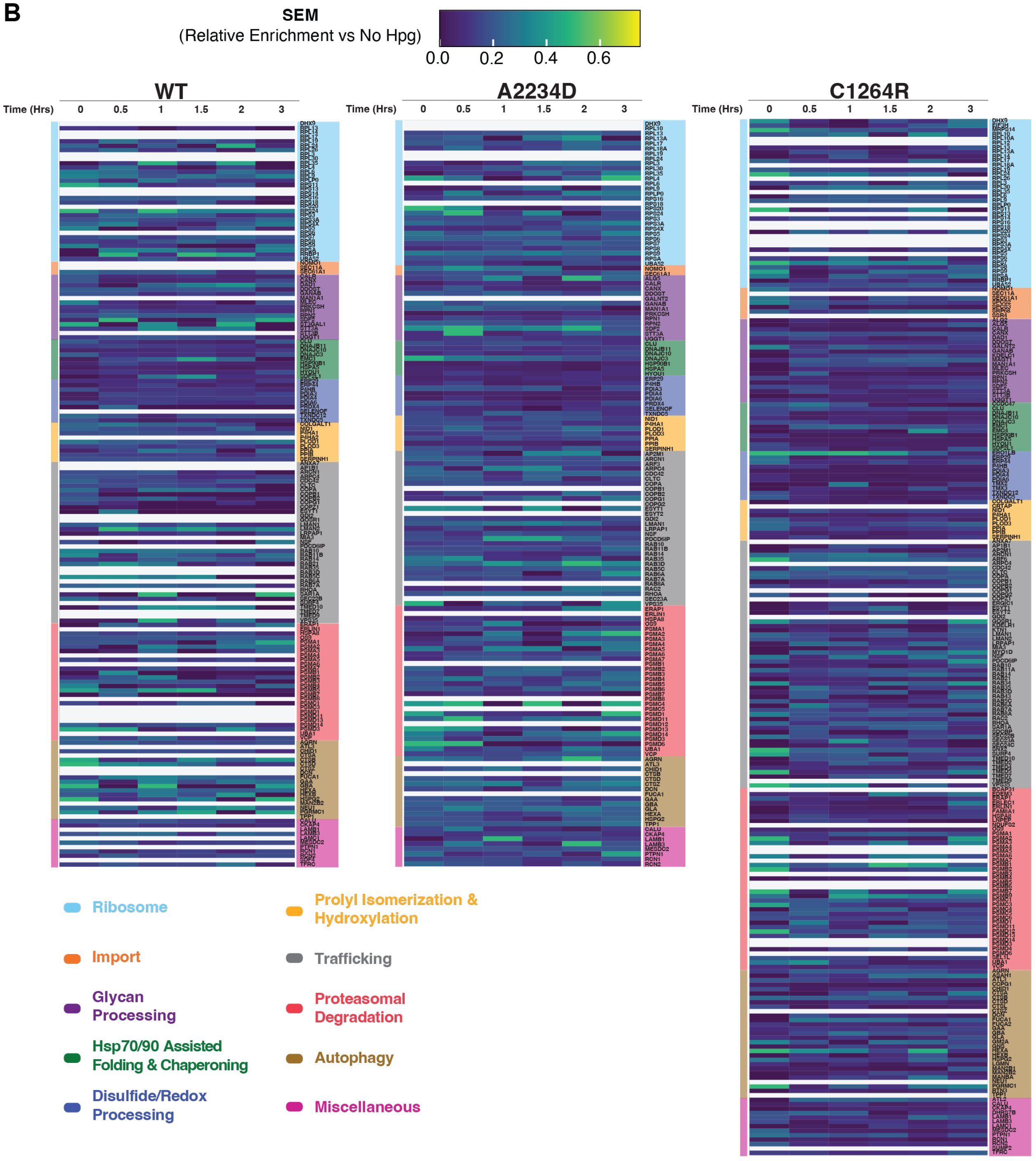
B
SEM
(Relative Enrichment vs No Hpg)
0.0
0.2
0.4
0.6
WT
A2234D
C1264R
Time (Hrs)
0
0.5
1
1.5
2
3
Ribosome
Import
Glycan Processing
Hsp70/90 Assisted Folding & Chaperoning
Disulfide/Redox Processing
Prolyl Isomerization & Hydroxylation
Trafficking
Proteasomal Degradation
Autophagy
Miscellaneous

Appendix Figure S4 – Summary of Tg TRIP Data –scaled heatmap
(A) Analysis showing the scaled log2 fold change enrichment of Tg interactors measured by TRIP for time-resolved analysis. Chase samples were pulse-labeled with Hpg (200µM) for 1 hour. Cells were harvested at specified time points and cross-linked with DSP (0.5mM) for 10 minutes to capture transient proteoastasis network interactions. Lysates were functionalized with TAMRA-Azide-PEG-Desthiobiotin probe CuAAC Click reaction. Chase samples were processed through the dual affinity purification TRIP workflow and processed for mass spectrometry. (-) Hpg samples were processed through the entire dual affinity purification TRIP workflow including 3-hour chase period, absent Hpg labeling, and used for enrichment analysis. Data were processed in R with custom scripts. TMT abundances across chase samples were normalized to Tg TMT abundance as described in the Materials and Methods section of the manuscript. For relative enrichment analysis, the means of log2 interaction differences were scaled to values from 0 to 1, where a value of 1 represented the time point at which the enrichment reached the maximum, while log2 values below the (-) Hpg condition were set to zero.
(B) Standard error of the mean (SEM) of the scaled log2 fold change enrichment of Tg interactors. n = 5 for WT and n= 6 for A2234D and C1264R.
Source data can be found in **Dataset EV4**. Script available at github.com/wrighmt1/2022_TRIP.

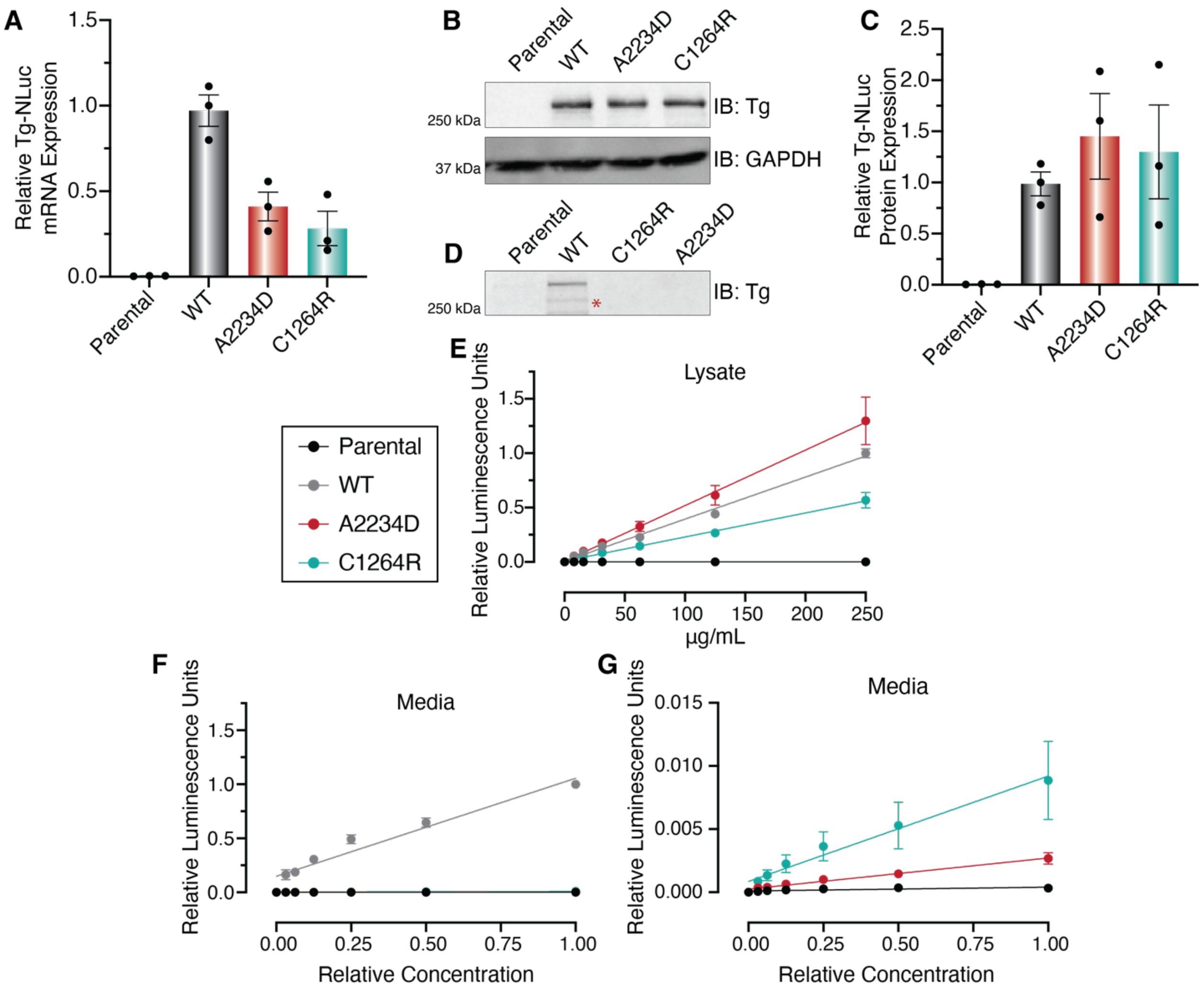

Appendix Figure S5 - Validation of Tg-NLuc stable cell lines

(A) Relative expression of Tg-NLuc RNA in engineered HEK293 cells measured by qRT-PCR. After transfections with Tg-FT pcDNA and flp recombinase pOG44, cells were placed under selection with Hygromycin B (100 µg/mL) to select site-specific recombinants. Resistant clonal lines were then screened for Tg-NLuc expression. Data was first normalized to a GAPDH loading control followed by normalization to median WT Tg-NLuc expression and represented as mean ± SEM. Primers for detection described in **Table EV2**.

(B) Western blot analysis of Tg-NLuc expression in engineered HEK293 cells in lysates. Tg-NLuc signal detected via Tg antibody. Tg is only detectable in isogenic cells co-transfected with Tg-NLuc pcDNA and flp recombinase pOG44, while Tg signal is absent in parental cells. GAPDH used as a loading control.

(C) Quantification of relative expression of Tg-NLuc in engineered HEK293 cells measured by western blot analysis in (B). Data first normalized to GAPDH used as a loading control, followed by normalization to median WT Tg-NLuc expression and represented as mean ± SEM.

(D) Western blot analysis of Tg-NLuc expression in media from engineered HEK293 cells. WT Tg-NLuc is efficiently secreted and detectable, while C1264R and A2234D secretion is drastically decreased compared to WT Tg-NLuc and is not detectable via western blot analysis.

(E-F) Standard curve exhibiting linearity of Tg-NLuc luminescence response based on total protein concentration of lysate (E) and media (F). Lysate or media from Tg-NLuc expressing

cells underwent serial dilutions prior to luminescence being measured with the nano-glo luciferase assay system (Promega, N1110). All samples exhibit a linear luminescence signal in response to substrate turnover, while luminescence signal is absent in parental cells. Data is normalized to median WT Tg-NLuc luminescence and represented as mean ± SEM.

(G) Rescaled standard curve from (F) exhibiting linearity of mutant Tg-NLuc luminescence response based on relative protein concentration in media. C1264R and A2234D secretion is drastically decreased compared to WT Tg-NLuc but still detectable above background (parental cells). Data is normalized to median WT Tg-NLuc luminescence and represented as mean ± SEM.

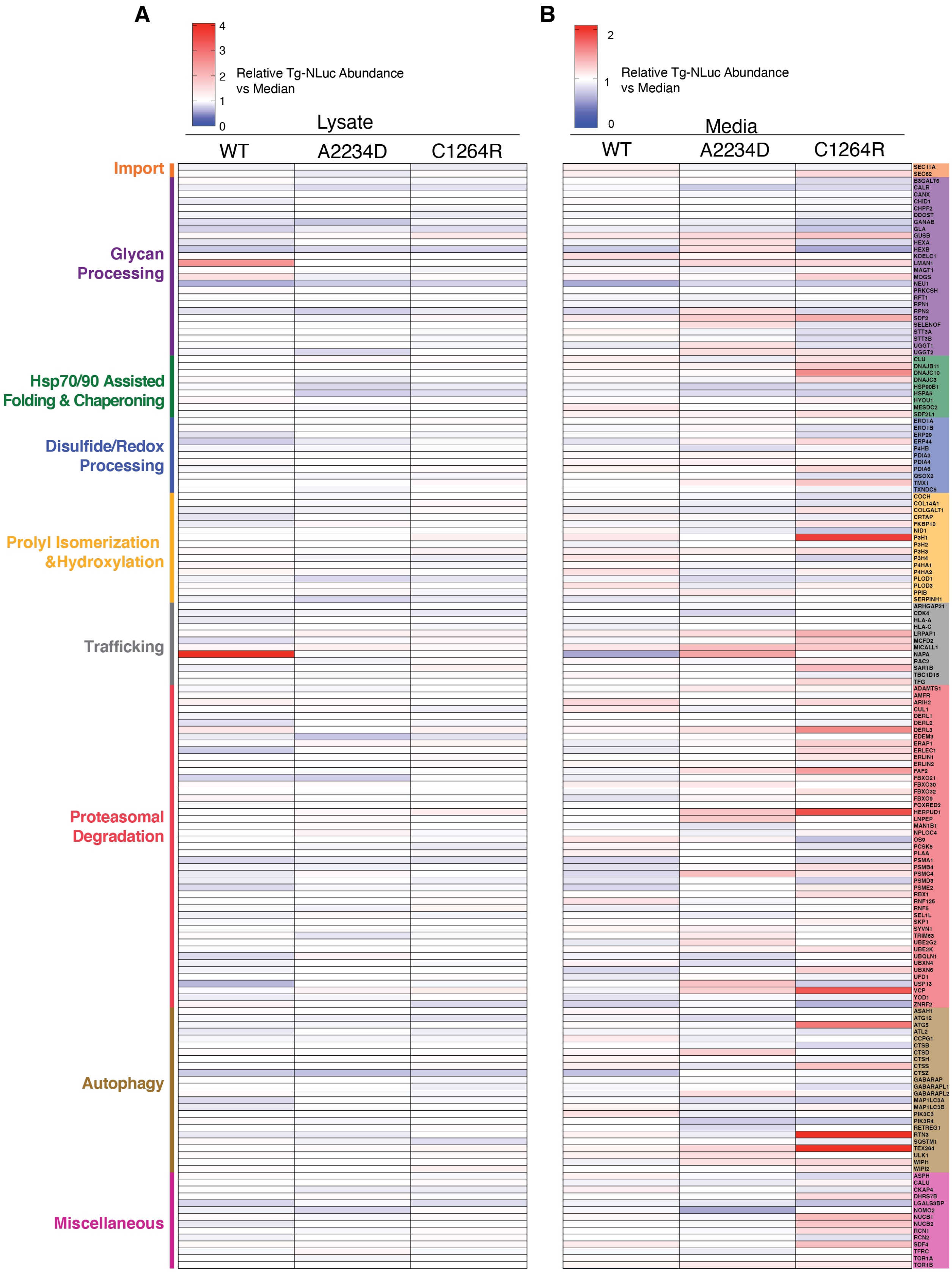
A
4
3
2
1
0
Relative Tg-NLuc Abundance vs Median
Lysate
WT
A2234D
C1264R
Import
Glycan Processing
Hsp70/90 Assisted Folding & Chaperoning
Disulfide/Redox Processing
Prolyl Isomerization &Hydroxylation
Trafficking
Proteasomal Degradation
Autophagy
Miscellaneous
B
2
1
0
Relative Tg-NLuc Abundance vs Median
Media
WT
A2234D
C1264R
SEC11A
SEC62
B3GALT6
CALR
CANX
CHID1
CHPF2
DDOST
GANAB
GLA
GUSB
HEXA
HEXB
KDELC1
LMAN1
MAGT1
MOGS
NEU1
PRKCSH
RFT1
RPN1
RPN2
SDF2
SELENOF
STT3A
STT3B
UGGT1
UGGT2
CLU
DNAJB11
DNAJC10
DNAJC3
HSP90B1
HSPA5
HYOU1
MESDC2
SDF2L1
ERO1A
ERO1B
ERP29
ERP44
P4HB
PDIA3
PDIA4
PDIA6
QSOX2
TMX1
TXNDC5
COCH
COL14A1
COLGALT1
CRTAP
FKBP10
NID1
P3H1
P3H2
P3H3
P3H4
P4HA1
P4HA2
PLOD1
PLOD3
PPIB
SERPINH1
ARHGAP21
CDK4
HLA-A
HLA-C
LRPAP1
MCFD2
MICALL1
NAPA
RAC2
SAR1B
TBC1D15
TFG
ADAMTS1
AMFR
ARIH2
CUL1
DERL1
DERL2
DERL3
EDEM3
ERAP1
ERLEC1
ERLIN1
ERLIN2
FAF2
FBXO21
FBXO30
FBXO32
FBXO9
FOXRED2
HERPUD1
LNPEP
MAN1B1
NPLOC4
OS9
PCSK5
PLAA
PSMA1
PSMB4
PSMC4
PSMD3
PSME2
RBX1
RNF125
RNF5
SEL1L
SKP1
SYVN1
TRIM63
UBE2G2
UBE2K
UBQLN1
UBXN4
UBXN6
UFD1
USP13
VCP
YOD1
ZNRF2
ASAH1
ATG12
ATG5
ATL2
CCPG1
CTSB
CTSD
CTSH
CTSS
CTSZ
GABARAP
GABARAPL1
GABARAPL2
MAP1LC3A
MAP1LC3B
PIK3C3
PIK3R4
RETREG1
RTN3
SQSTM1
TEX264
ULK1
WIPI1
WIPI2
ASPH
CALU
CKAP4
DHRS7B
LGALS3BP
NOMO2
NUCB1
NUCB2
RCN1
RCN2
SDF4
TFRC
TOR1A
TOR1B

Appendix Figure S6 - Summary of siRNA screening data

(A) Analysis showing the relative Tg-NLuc abundance changes in lysate with siRNA knockdown of select genes. Approximately 36 hours after transfection with 25nM siRNAs cells were replenished with fresh media and Tg-NLuc abundance in lysate was measure after 4 hours using the nano-glo luciferase assay system. Data was median normalized across individual 96-well plates (Chung et al., 2008). Data represents two independent experiments for WT-NLuc and A2234D-NLuc, and three independent experiments for C1264R NLuc. Cutoff criteria for hits were set to those genes that increased or decreased Tg-NLuc abundance in lysate or media by 3σ.

(B) Analysis showing the relative Tg-NLuc abundance changes in media with siRNA knockdown of select genes. Approximately 36 hours after transfection with 25nM siRNAs cells were replenished with fresh media and Tg-NLuc abundance in media was measure after 4 hours using the nano-glo luciferase assay system. Data was median normalized across individual 96-well plates (Chung et al., 2008). Data represents two independent experiments for WT-NLuc and A2234D-NLuc, and three independent experiments for C1264R NLuc. Cutoff criteria for hits were set to those genes that increased or decreased Tg-NLuc abundance in lysate or media by 3σ. Source data can be found in **Dataset EV5**.

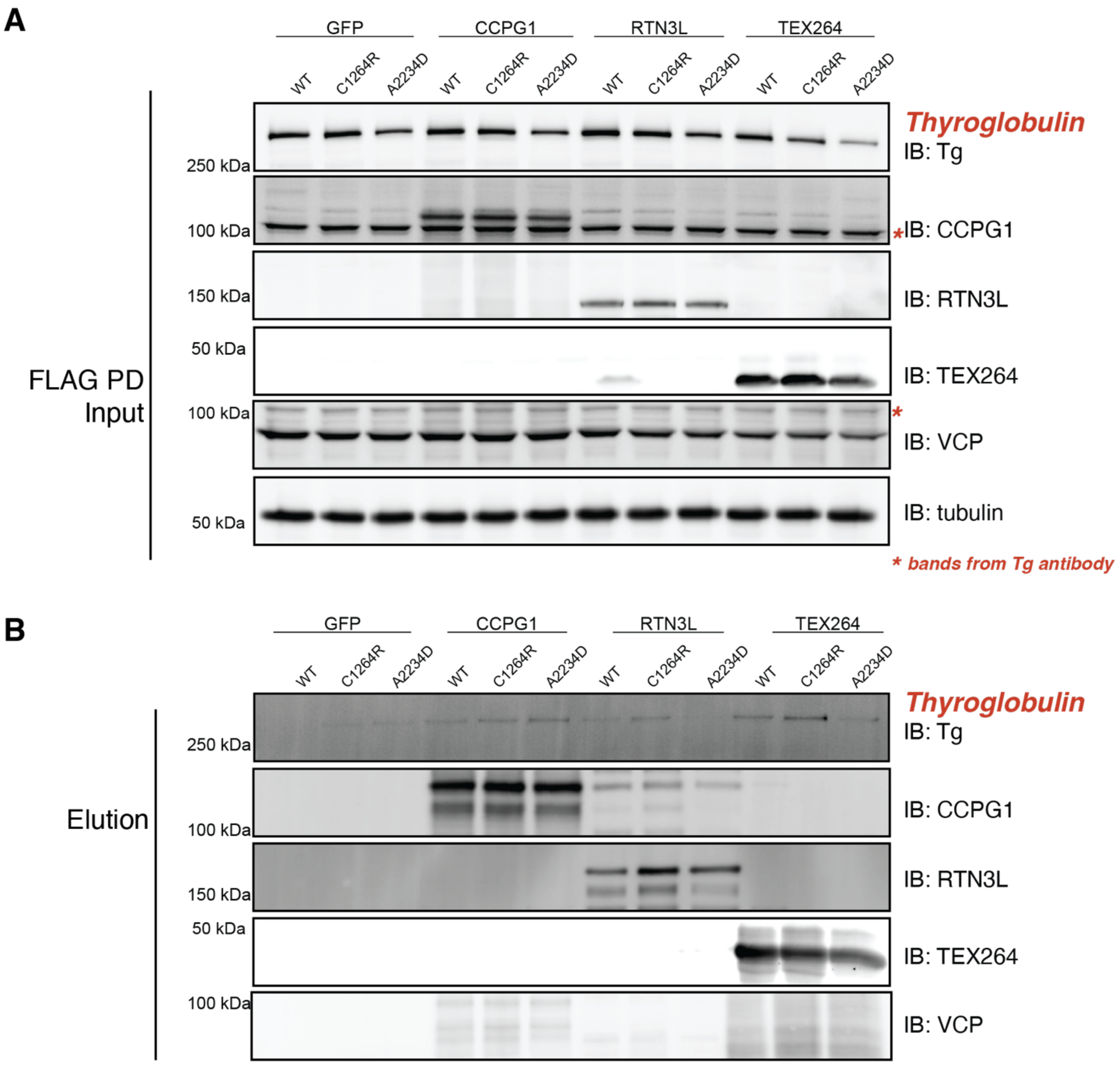

Appendix Figure S7 - Mutant Tg shows selective enrichment with TEX264 in a screen of multiple ER-phagy receptors
(A) Western blot of FLAG-tagged ER-phagy receptors in cells expressing Tg.
(B) FLAG co-IP elution samples. Western blot shows enrichment of ER-phagy receptor after pulldown (IB: CCPG1, RTN3L, TEX264). Selective enrichment of mutant Tg is present with TEX264 expression. Some Tg enrichment is also detectable with CCPG1 and RTN3L. In contrast, VCP shows no interaction with TEX264, suggesting that its interaction with Tg may be independent of ER-phagy receptors.

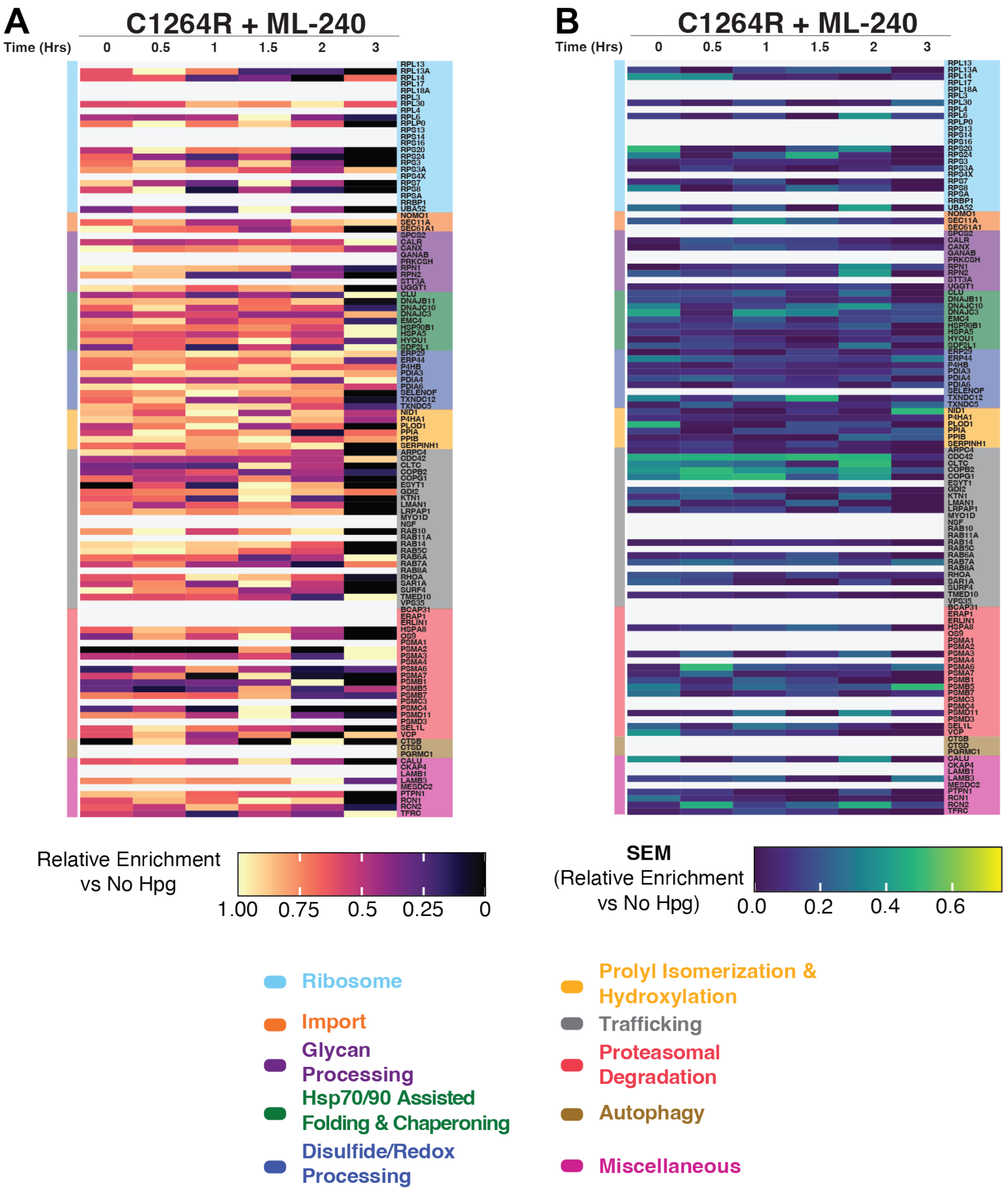

Appendix Figure S8 - TRIP of C1264R Tg-FT FRT cells with pharmacological VCP inhibition
(A) Analysis showing the scaled log2 fold change enrichment of C1264R Tg interactors measured by TRIP with ML-240 treatment. Chase samples were pulse labeled with Hpg (200 µM final concentration) in the presence of ML-240 (10 µM) for 1 h. Cells were dosed with ML-240 (10µM) throughout the chase period, harvested at specified time points and cross linked with DSP (0.5mM) for 10 minutes to capture transient proteoastasis network interactions. Lysates were functionalized with TAMRA-Azide-PEG-Desthiobiotin probe using copper catalyzed azide-alkyne cycloaddition (CuAAC). Chase samples were processed through the dual affinity purification TRIP workflow and processed for mass spectrometry. (-) Hpg samples were processed through the entire dual affinity purification TRIP workflow in the presence of

ML-240 (10μM) including 3 h chase period, absent Hpg labeling, and used for enrichment analysis.
Data were processed in R with custom scripts. TMT abundances across chase samples were normalized to Tg TMT abundance as described within the Materials and Methods section of the manuscript. For relative enrichment analysis, the means of log2 interaction differences were scaled to values from 0 to 1, where a value of 1 represented the time point at which the enrichment reached the maximum, while log2 values below the (-) Hpg condition were set to zero.
(B) Analysis showing the SEM of the scaled log2 fold change enrichment of C1264R Tg interactors measured by TRIP with ML-240 treatment. Chase and (-) Hpg samples were processed as described above in (A). Data were processed in R with custom scripts. TMT abundances across chase samples were normalized to Tg TMT abundance as described within the Materials and Methods section of the manuscript. Standard error of the mean (SEM) was then calculated from these enrichment values to examine the reproducibility of these measurements. Script available at github.com/wrighmt1/2022_TRIP. Source data for (A) – (B) can be found in **Dataset EV4.**
