# Supplementary material for: Time-resolved interactome profiling deconvolutes secretory protein quality control dynamics
Source: Mol Syst Biol. 2024 Aug 5;20(9):1049–75. doi: 10.1038/s44320-024-00058-1 (PMC11369088; doi:10.1038/s44320-024-00058-1)
Supplement: Supplementary file 11 — Source data Fig. 1 [file 44320_2024_58_MOESM11_ESM.zip › Figure 1/1C/Fig 1C - Biotin PD Inputs - KDEL [HSP90B1 & HSPA5] (StarBright B700).pdf]

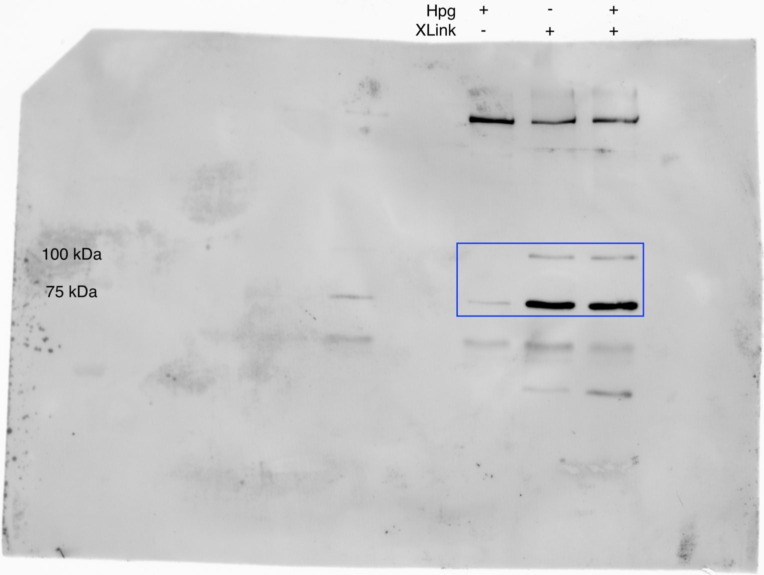
Hpg + - +
XLink - + +
100 kDa
75 kDa
