# Supplementary figures and images for: Time-resolved interactome profiling deconvolutes secretory protein quality control dynamics
Source: Mol Syst Biol. 2024 Aug 5;20(9):1049–75. doi: 10.1038/s44320-024-00058-1 (PMC11369088; doi:10.1038/s44320-024-00058-1)

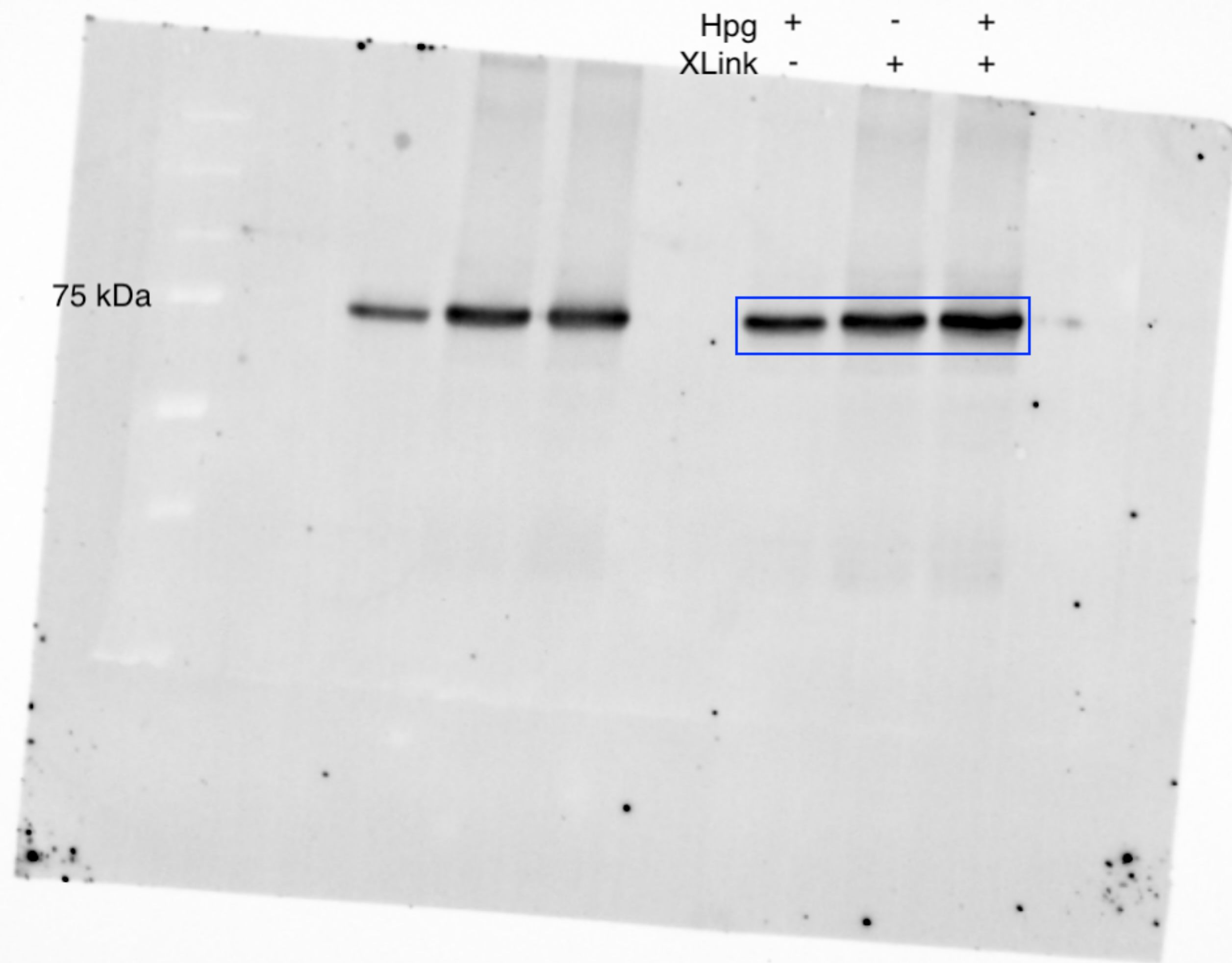

Supplement: Supplementary file 11 — Source data Fig. 1 [file 44320_2024_58_MOESM11_ESM.zip › Figure 1/1B/Fig 1B - FLAG IP Inputs - PDIA4 (IRDye 800CW).pdf]

Hpg + - +  
XLink - + +

37 kDa

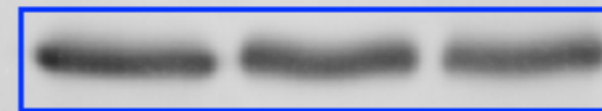

Supplement: Supplementary file 11 — Source data Fig. 1 [file 44320_2024_58_MOESM11_ESM.zip › Figure 1/1B/Fig 1B - FLAG IP Inputs - GAPDH (StarBright B700).pdf]

250 kDa

|       |   |   |   |
|-------|---|---|---|
| Hpg   | + | - | + |
| XLink | - | + | + |

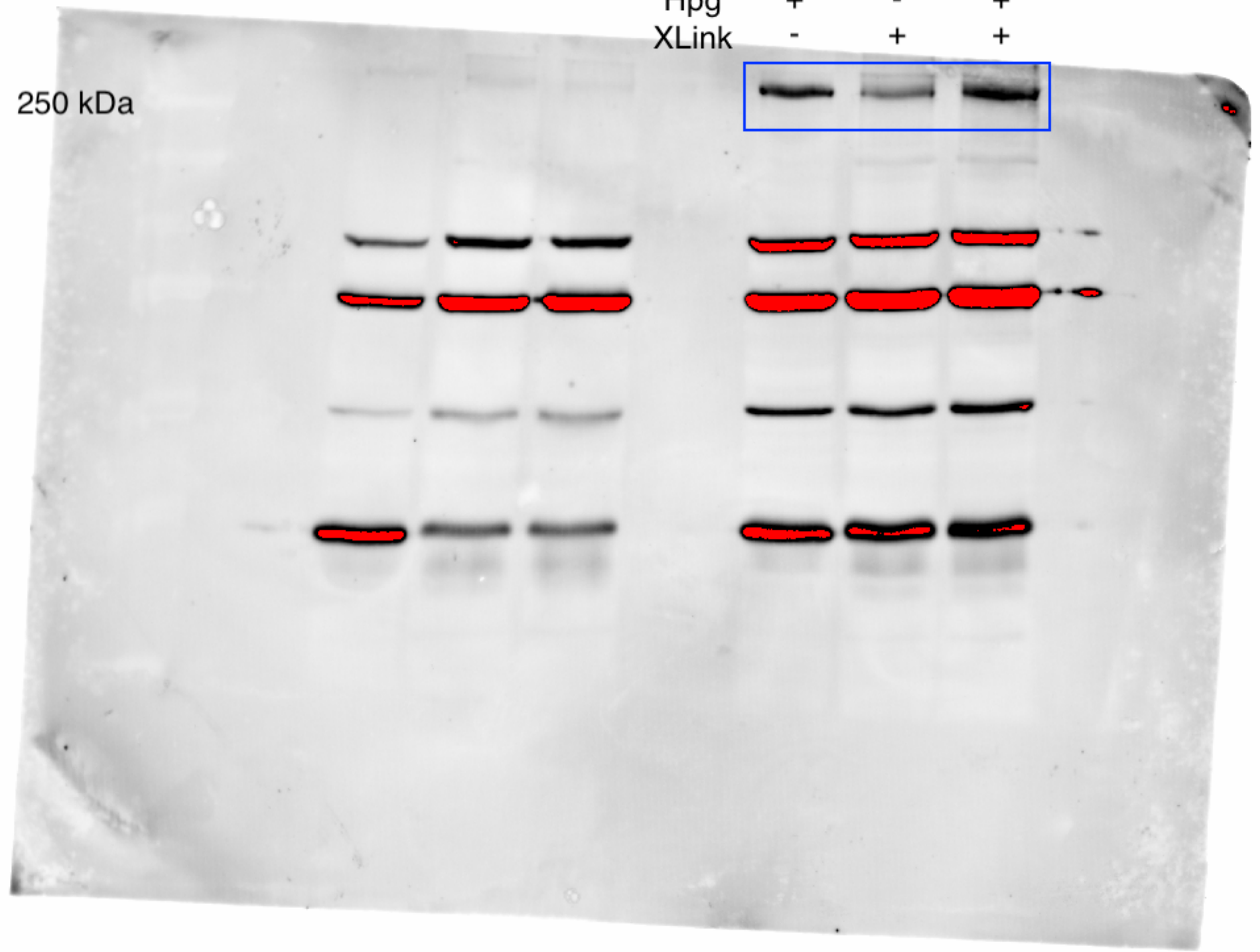

Supplement: Supplementary file 11 — Source data Fig. 1 [file 44320_2024_58_MOESM11_ESM.zip › Figure 1/1B/Fig 1B - FLAG IP Input - M2 [FLAG] (StarBright B700).pdf]

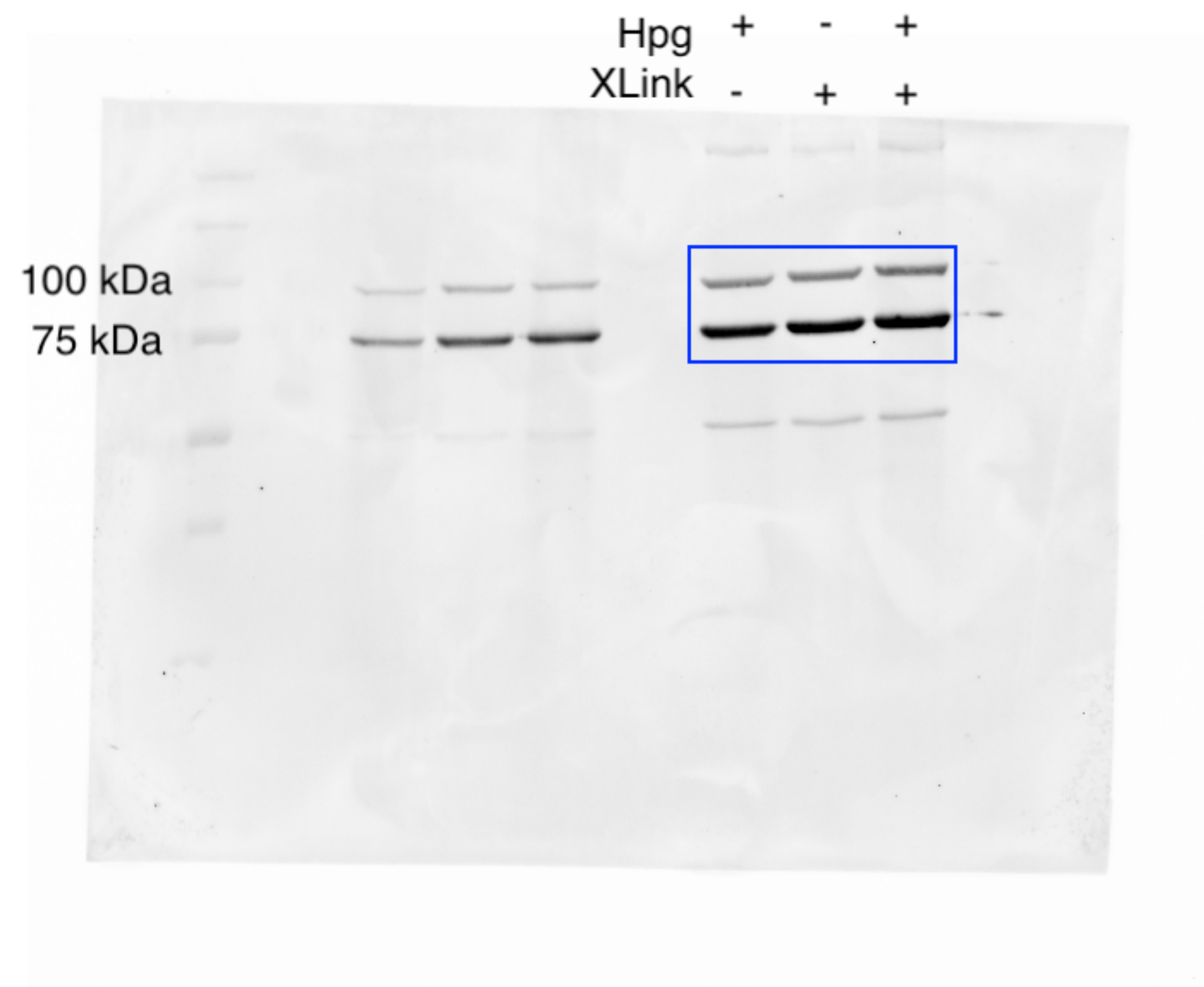

Supplement: Supplementary file 11 — Source data Fig. 1 [file 44320_2024_58_MOESM11_ESM.zip › Figure 1/1B/Fig 1B - FLAG IP Inputs - KDEL [HSP90B1 & HSPA5] (StarBright B700).pdf]

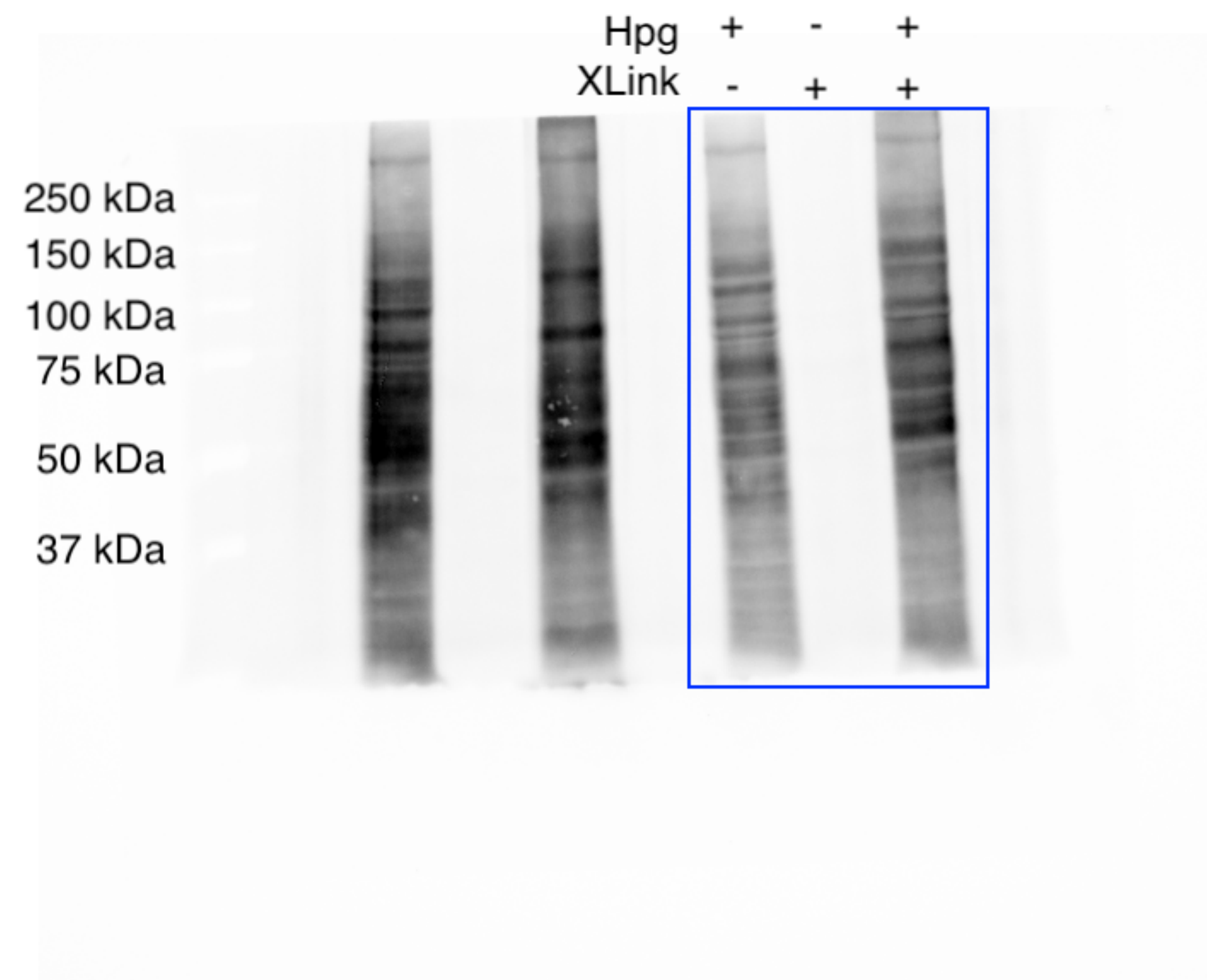

Supplement: Supplementary file 11 — Source data Fig. 1 [file 44320_2024_58_MOESM11_ESM.zip › Figure 1/1B/Fig 1B - FLAG IP Inputs - TAMRA Probe (Rhodamine).pdf]

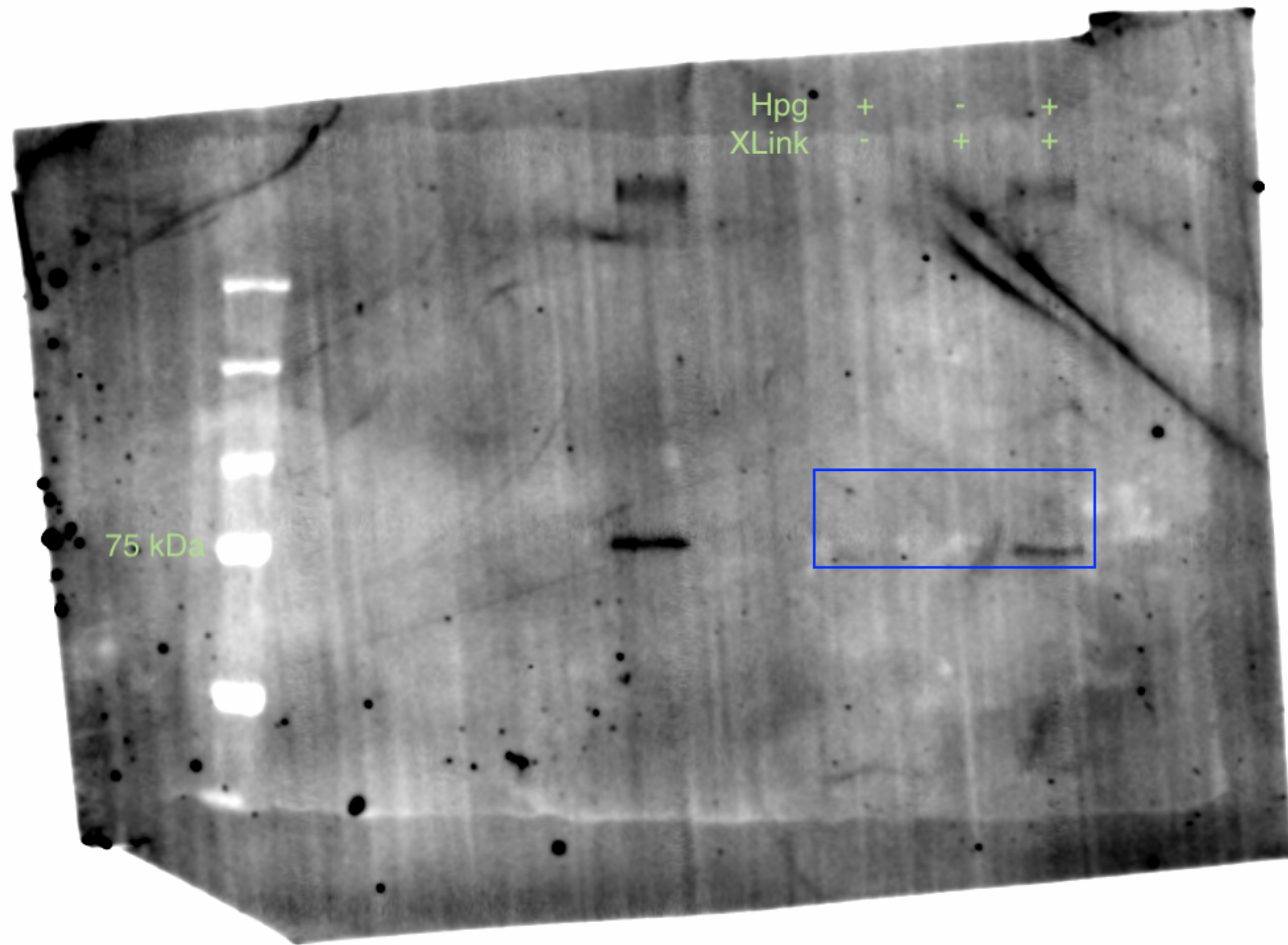

Supplement: Supplementary file 11 — Source data Fig. 1 [file 44320_2024_58_MOESM11_ESM.zip › Figure 1/1D/Fig 1D - Biotin PD Elutions - PDIA4 (IRDye 800CW).pdf]

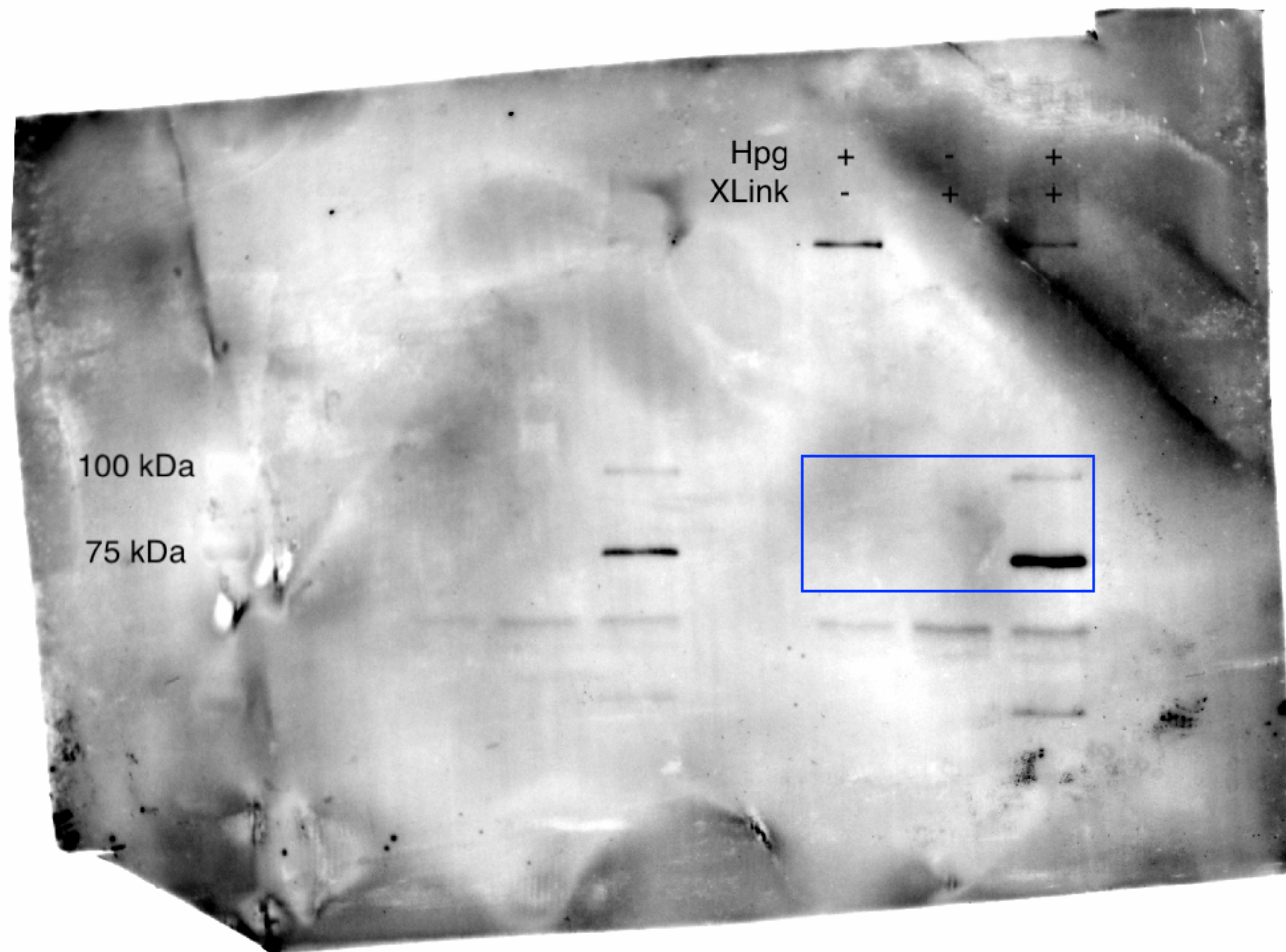

Supplement: Supplementary file 11 — Source data Fig. 1 [file 44320_2024_58_MOESM11_ESM.zip › Figure 1/1D/Fig 1D - Biotin PD Elutions - KDEL [HSP90B1 & HSPA5] (StarBright B700).pdf]

250 kDa

| Hpg   | + | - | + |
|-------|---|---|---|
| XLink | - | + | + |

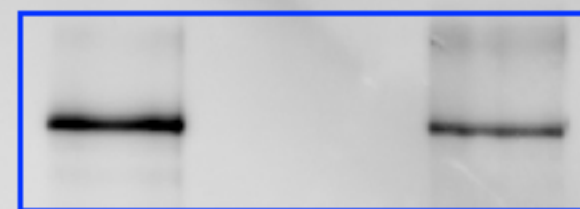

Supplement: Supplementary file 11 — Source data Fig. 1 [file 44320_2024_58_MOESM11_ESM.zip › Figure 1/1D/Fig 1D - Biotin PD Elutions - M2 [FLAG] (StarBright B700).pdf]

250 kDa

|       |   |   |   |
|-------|---|---|---|
| Hpg   | + | - | + |
| XLink | - | + | + |

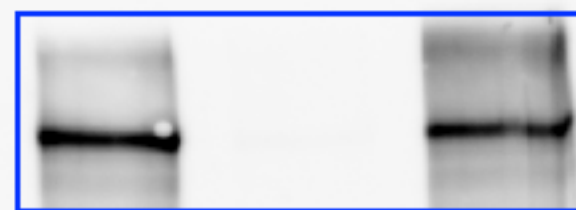

Supplement: Supplementary file 11 — Source data Fig. 1 [file 44320_2024_58_MOESM11_ESM.zip › Figure 1/1D/Fig 1D - Biotin PD Elutions - TAMRA Probe (Rhodamine).pdf]

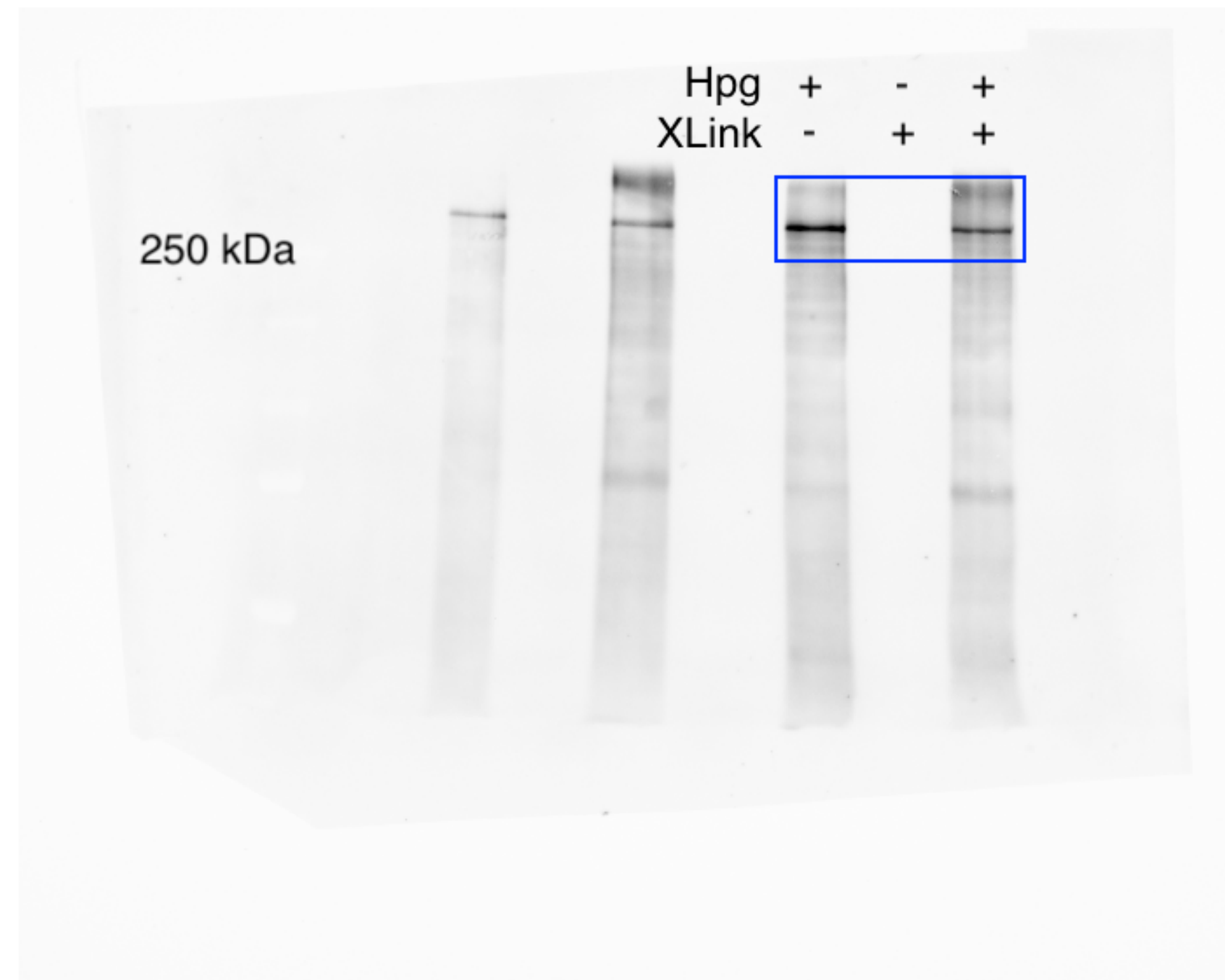

Supplement: Supplementary file 11 — Source data Fig. 1 [file 44320_2024_58_MOESM11_ESM.zip › Figure 1/1C/Fig 1C - Biotin PD Inputs - TAMRA Probe (Rhodamine).pdf]

|       |   |   |   |
|-------|---|---|---|
| Hpg   | + | - | + |
| XLink | - | + | + |

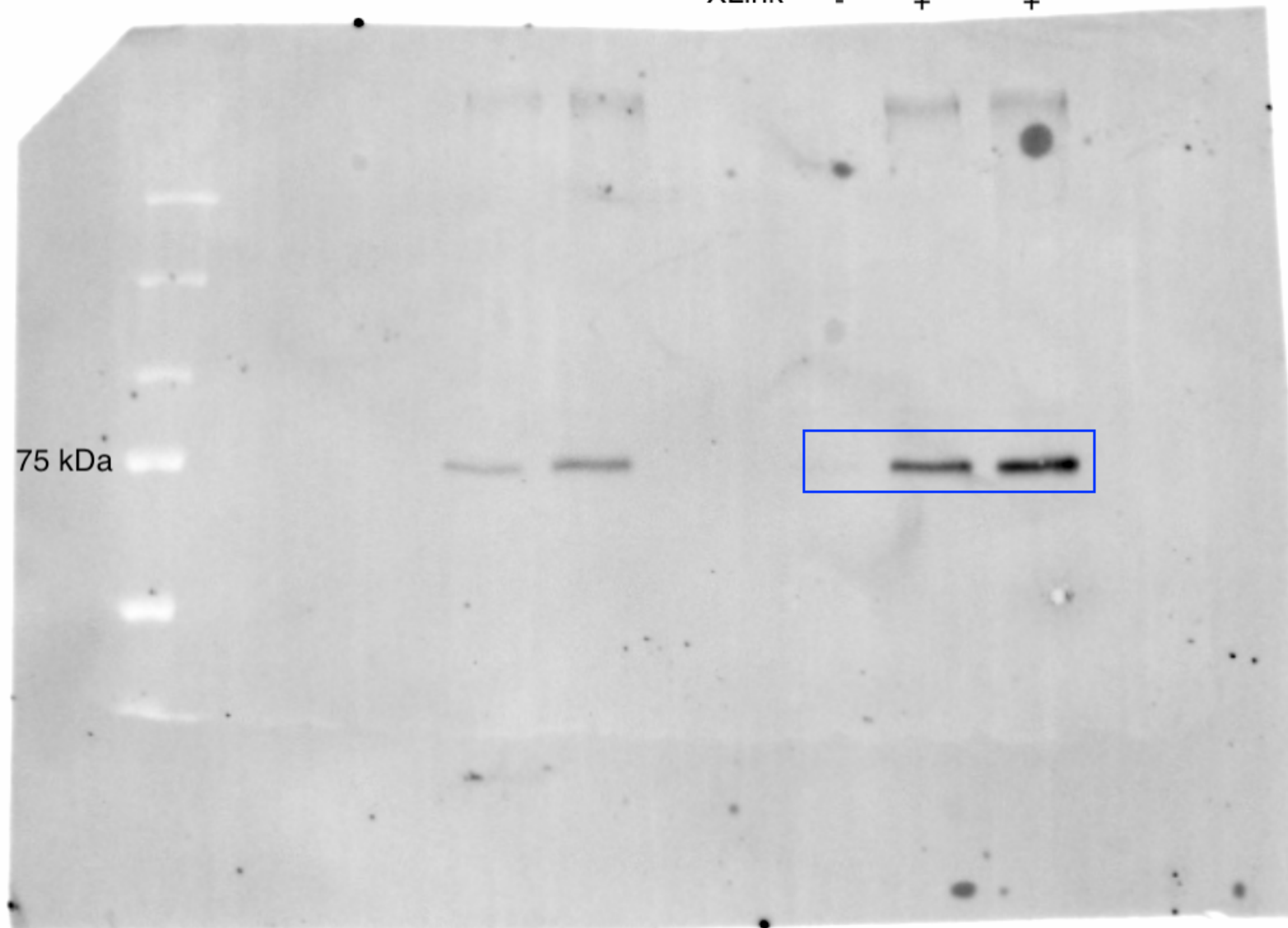

Supplement: Supplementary file 11 — Source data Fig. 1 [file 44320_2024_58_MOESM11_ESM.zip › Figure 1/1C/Fig 1C - Biotin PD Inputs - PDIA4 (IRDye 800CW).pdf]

|       |   |   |   |
|-------|---|---|---|
| Hpg   | + | - | + |
| XLink | - | + | + |

250 kDa

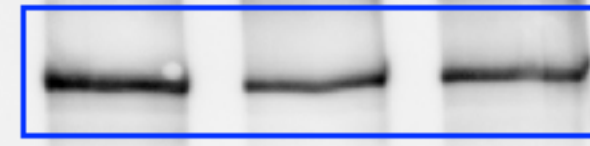

Supplement: Supplementary file 11 — Source data Fig. 1 [file 44320_2024_58_MOESM11_ESM.zip › Figure 1/1C/Fig 1C - Biotin PD Inputs - M2 [FLAG] (StarBright B700).pdf]

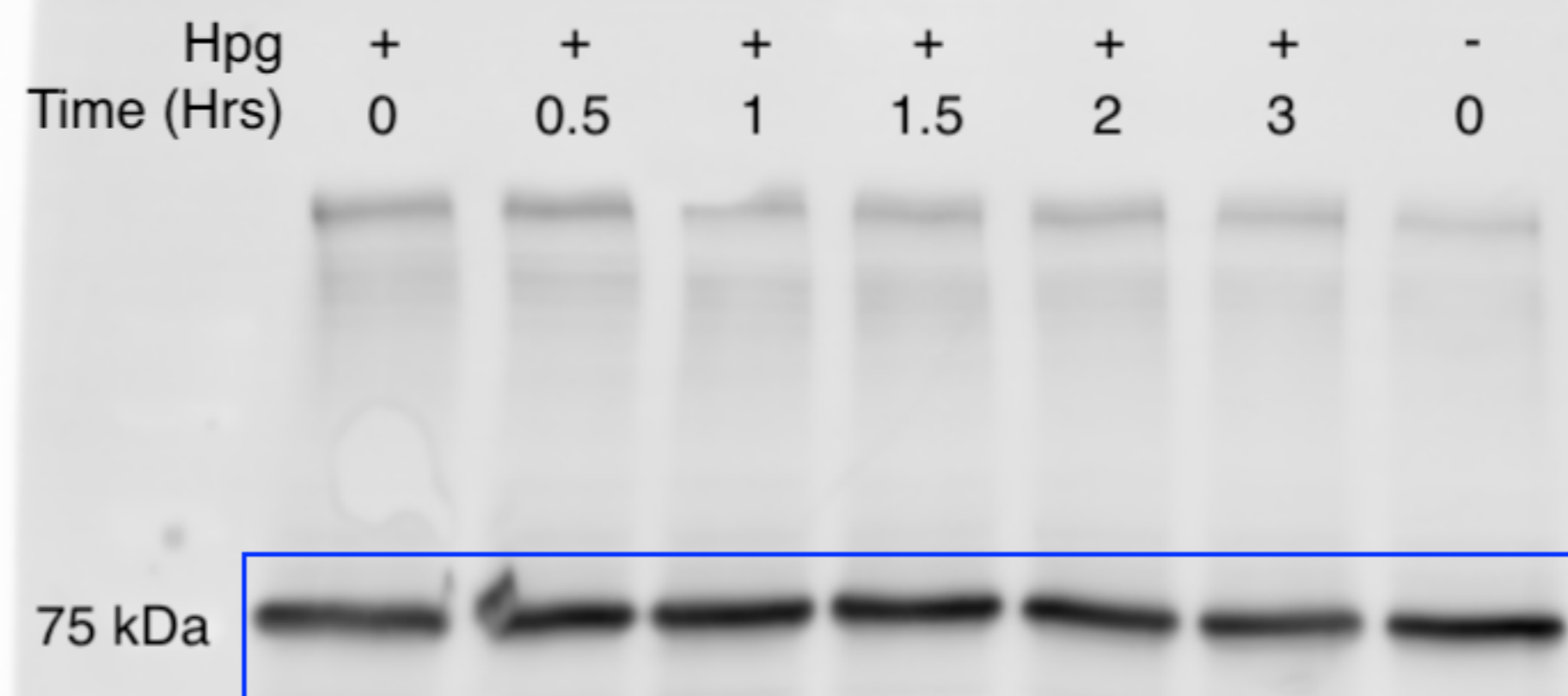

Supplement: Supplementary file 12 — Source data Fig. 2 [file 44320_2024_58_MOESM12_ESM.zip › Figure 2/2C/Fig 2C - FLAG IP Inputs - PDIA4 (IRDye 800CW).pdf]

| Hpg        | + | +   | + | +   | + | + | - |
|------------|---|-----|---|-----|---|---|---|
| Time (Hrs) | 0 | 0.5 | 1 | 1.5 | 2 | 3 | 0 |

100 kDa

75 kDa

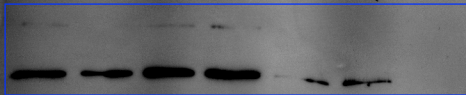

Supplement: Supplementary file 12 — Source data Fig. 2 [file 44320_2024_58_MOESM12_ESM.zip › Figure 2/2C/Fig 2C - Biotin PD Elution - KDEL [HSP90B1 & HSPA5] (StarBright B700).pdf]

| Hpg        | + | +   | + | +   | + | + | - |
|------------|---|-----|---|-----|---|---|---|
| Time (Hrs) | 0 | 0.5 | 1 | 1.5 | 2 | 3 | 0 |

250 kDa

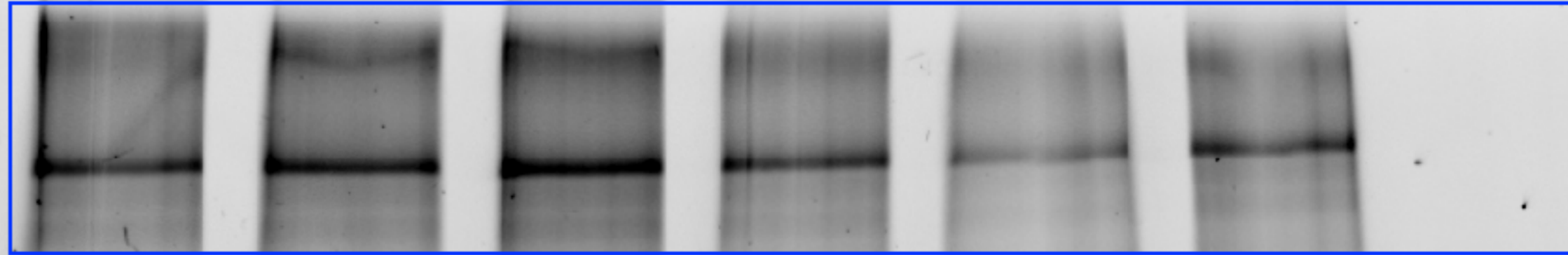

Supplement: Supplementary file 12 — Source data Fig. 2 [file 44320_2024_58_MOESM12_ESM.zip › Figure 2/2C/Fig 2C - Biotin PD Elution - Gel - TAMRA Probe (Rhodamine).pdf]

| Hpg        | + | +   | + | +   | + | + | - |
|------------|---|-----|---|-----|---|---|---|
| Time (Hrs) | 0 | 0.5 | 1 | 1.5 | 2 | 3 | 0 |

250 kDa

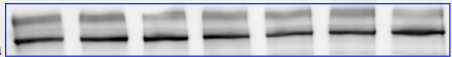

Supplement: Supplementary file 12 — Source data Fig. 2 [file 44320_2024_58_MOESM12_ESM.zip › Figure 2/2C/Fig 2C - FLAG IP Inputs - M2 [FLAG] (StarBright B700).pdf]

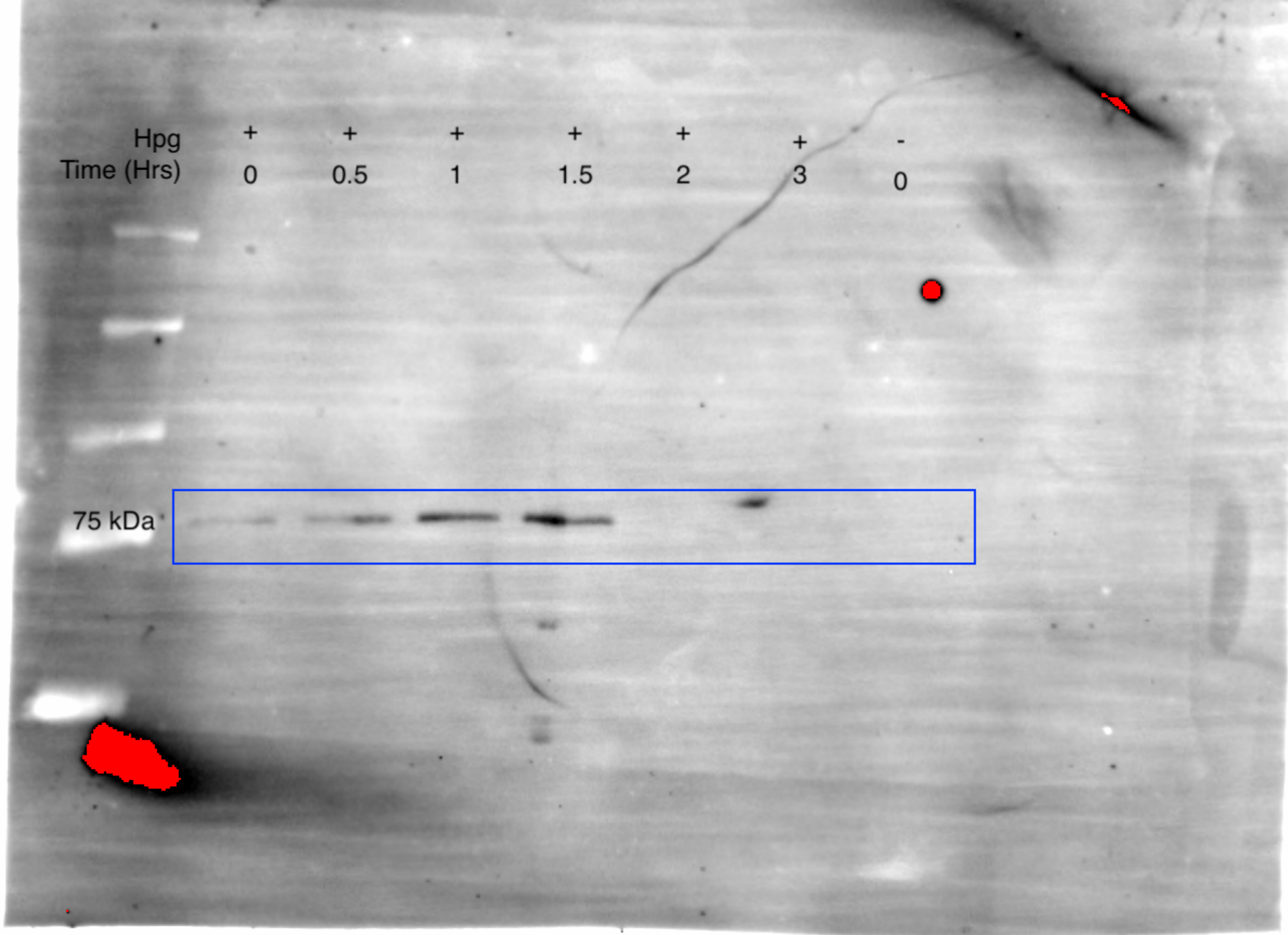

Supplement: Supplementary file 12 — Source data Fig. 2 [file 44320_2024_58_MOESM12_ESM.zip › Figure 2/2C/Fig 2C - Biotin PD Elution - PDIA4 (IRDye 800CW).pdf]

Hpg  
Time (Hrs)

+

0

+

0.5

+

1

+

1.5

+

2

+

3

-

0

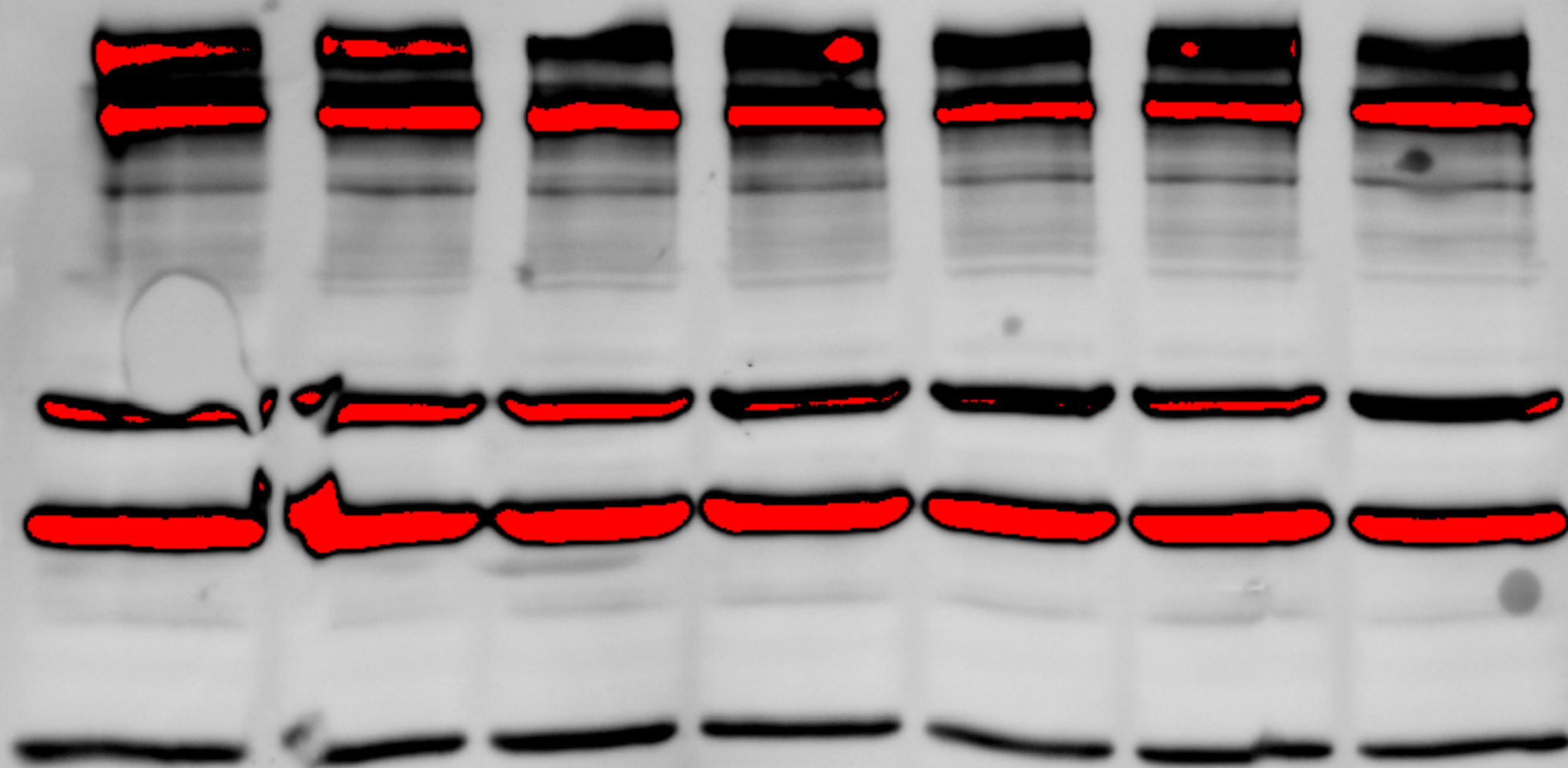

37 kDa

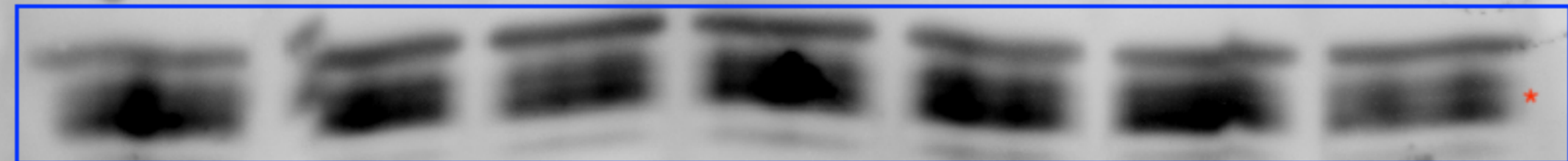

Supplement: Supplementary file 12 — Source data Fig. 2 [file 44320_2024_58_MOESM12_ESM.zip › Figure 2/2C/Fig 2C - FLAG IP Inputs - GAPDH (StarBright B700).pdf]

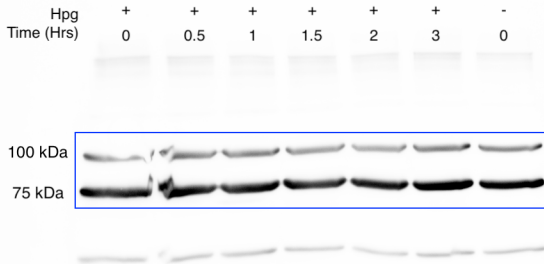

Supplement: Supplementary file 12 — Source data Fig. 2 [file 44320_2024_58_MOESM12_ESM.zip › Figure 2/2C/Fig 2C - FLAG IP Inputs - KDEL [HSP90B1 & HSPA5] (StarBright B700).pdf]

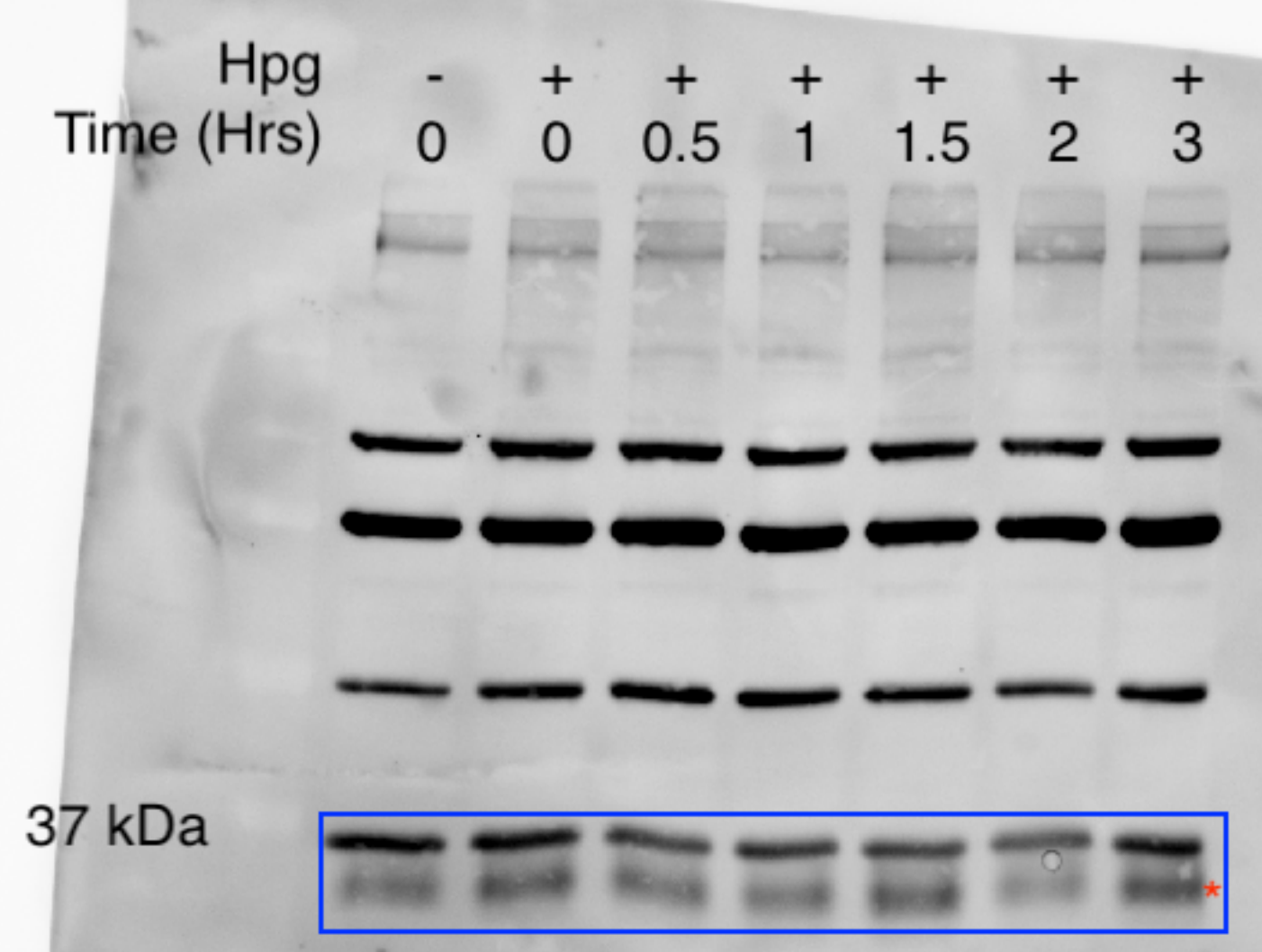

Supplement: Supplementary file 12 — Source data Fig. 2 [file 44320_2024_58_MOESM12_ESM.zip › Figure 2/2B/Fig 2B - FLAG IP Input - GAPDH (StarBright B700).pdf]

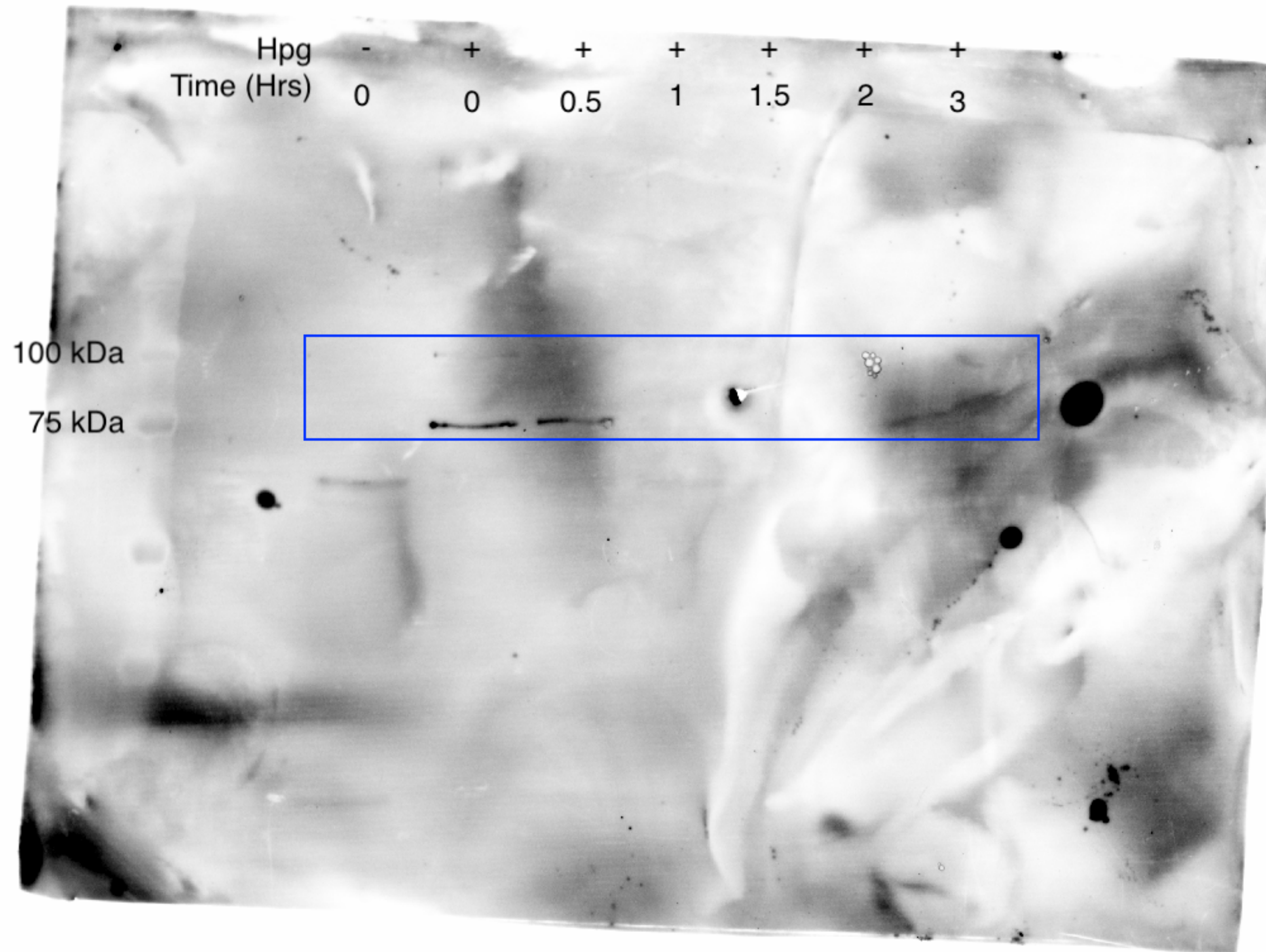

Supplement: Supplementary file 12 — Source data Fig. 2 [file 44320_2024_58_MOESM12_ESM.zip › Figure 2/2B/Fig 2B - BPD Elution - KDEL [HSP90B1 & HSPA5] (StarBright B700).pdf]

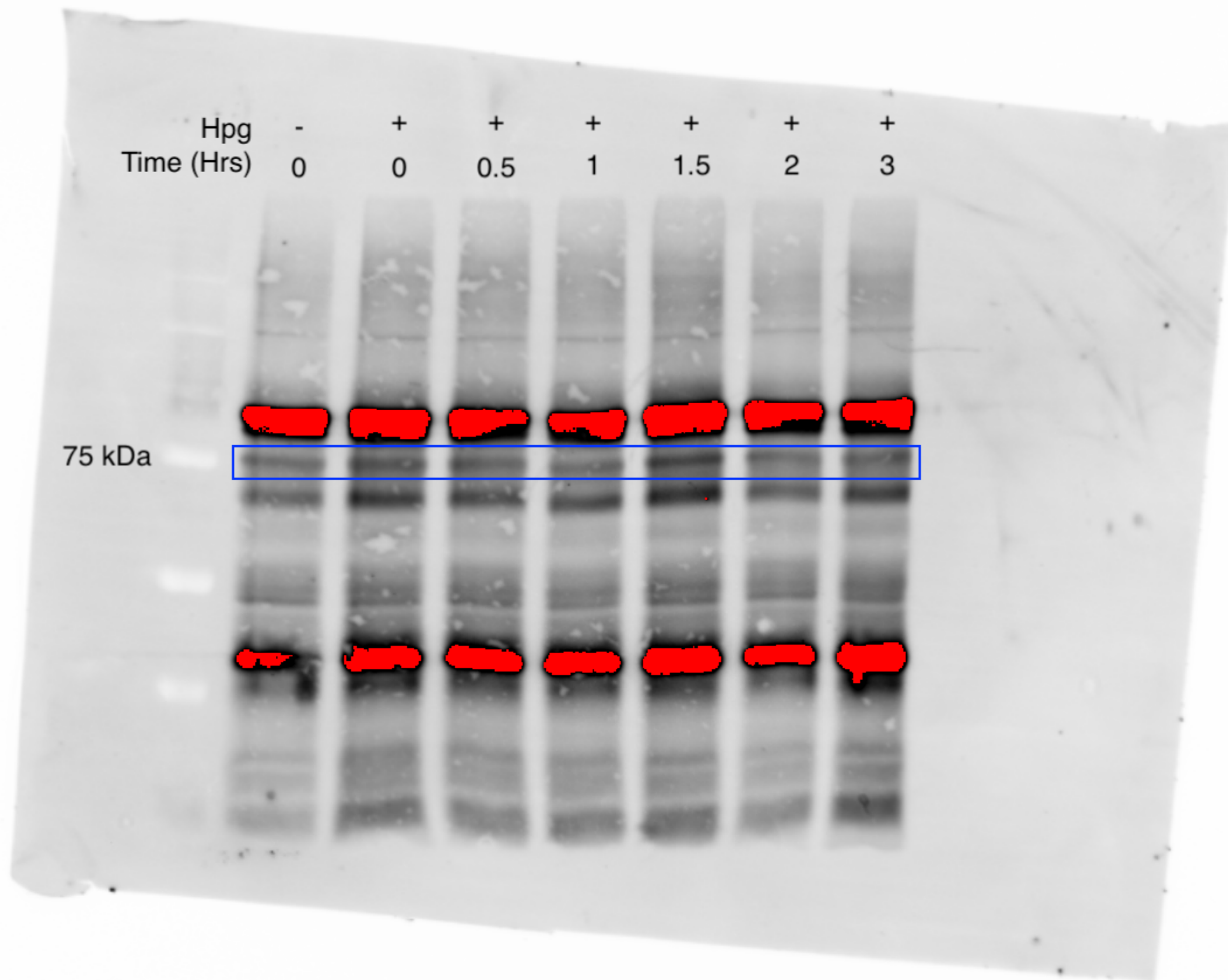

Supplement: Supplementary file 12 — Source data Fig. 2 [file 44320_2024_58_MOESM12_ESM.zip › Figure 2/2B/Fig 2B - FLAG IP Input - PDIA4 (IRDye 800CW).pdf]

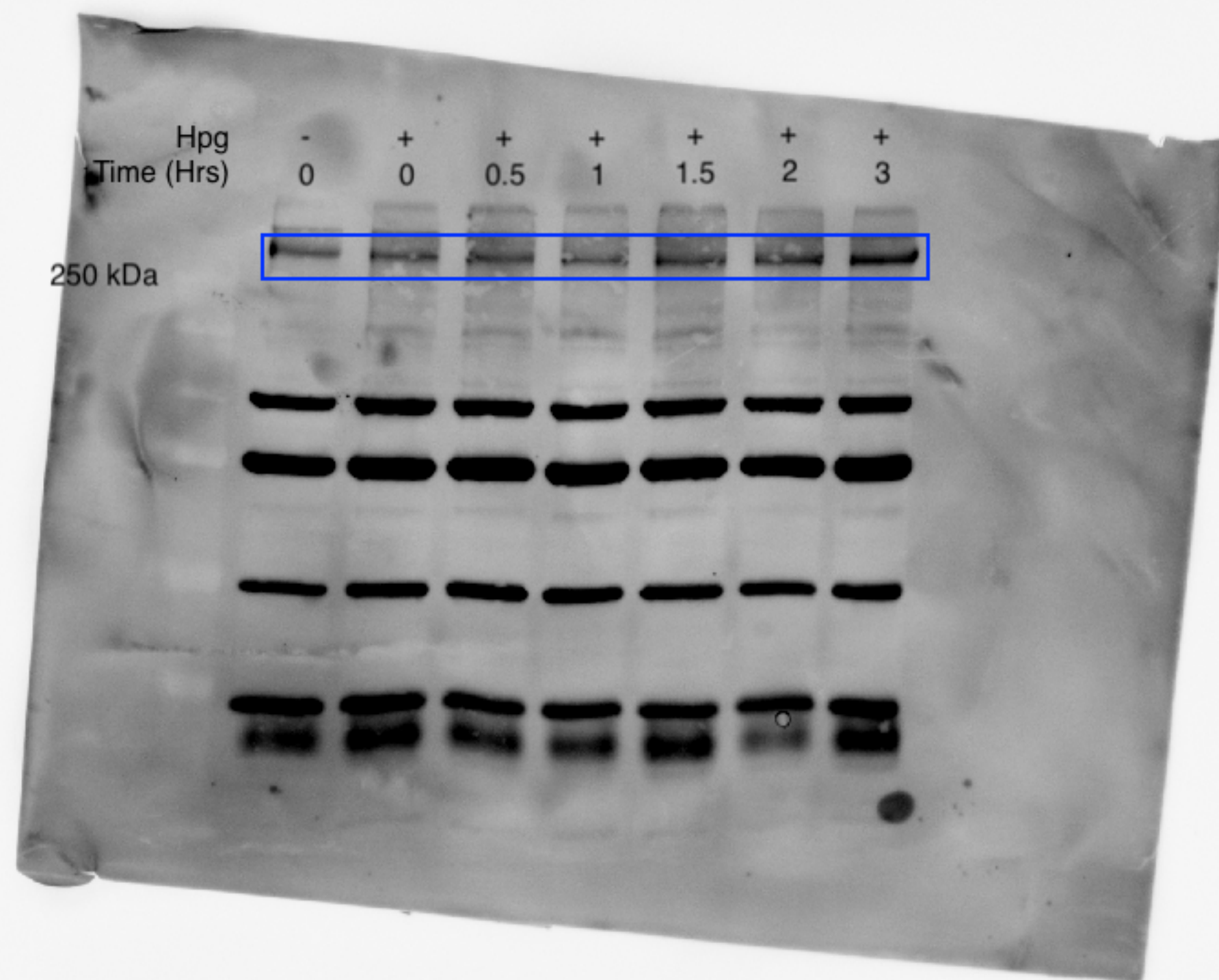

Supplement: Supplementary file 12 — Source data Fig. 2 [file 44320_2024_58_MOESM12_ESM.zip › Figure 2/2B/Fig 2B - FLAG IP Input - M2 [FLAG] (StarBright B700).pdf]

| Hpg        | - | + | +   | + | +   | + | + |
|------------|---|---|-----|---|-----|---|---|
| Time (Hrs) | 0 | 0 | 0.5 | 1 | 1.5 | 2 | 3 |

250 kDa

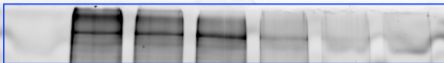

Supplement: Supplementary file 12 — Source data Fig. 2 [file 44320_2024_58_MOESM12_ESM.zip › Figure 2/2B/Fig 2B - BPD Elution - Gel - TAMRA Probe (Rhodamine).pdf]

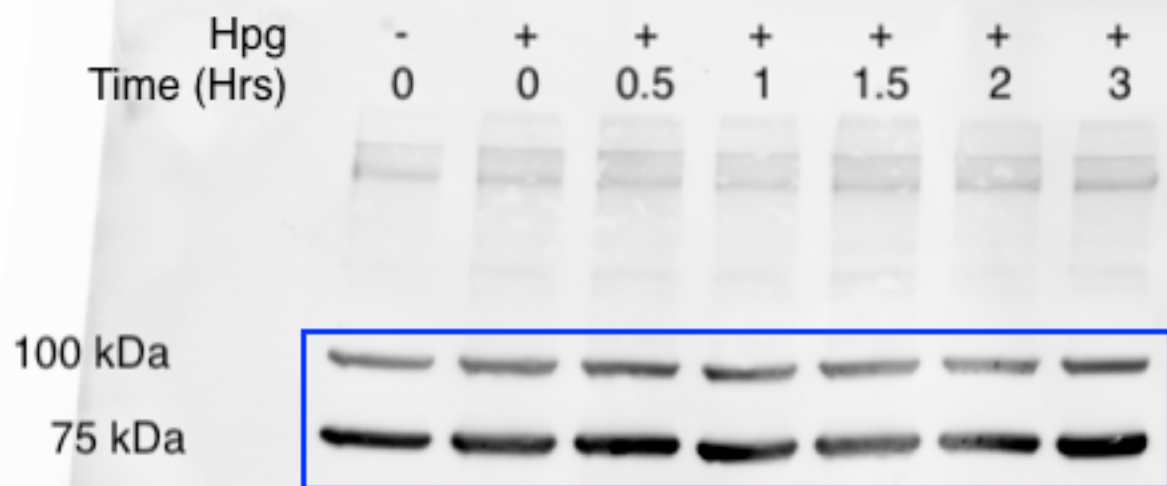

Supplement: Supplementary file 12 — Source data Fig. 2 [file 44320_2024_58_MOESM12_ESM.zip › Figure 2/2B/Fig 2B - FLAG IP Input - KDEL [HSP90B1 & HSPA5] (StarBright B700).pdf]

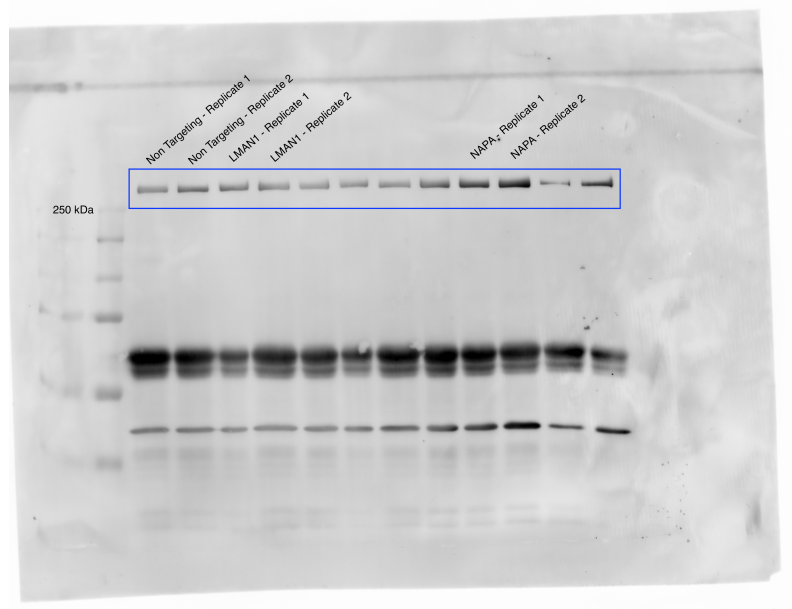

Supplement: Supplementary file 14 — Source data Fig. 5 [file 44320_2024_58_MOESM14_ESM.zip › Figure 5/5A/Fig 5A - Lysate - M2 [FLAG] (StarBright B700) - Replicates 1&2.pdf]

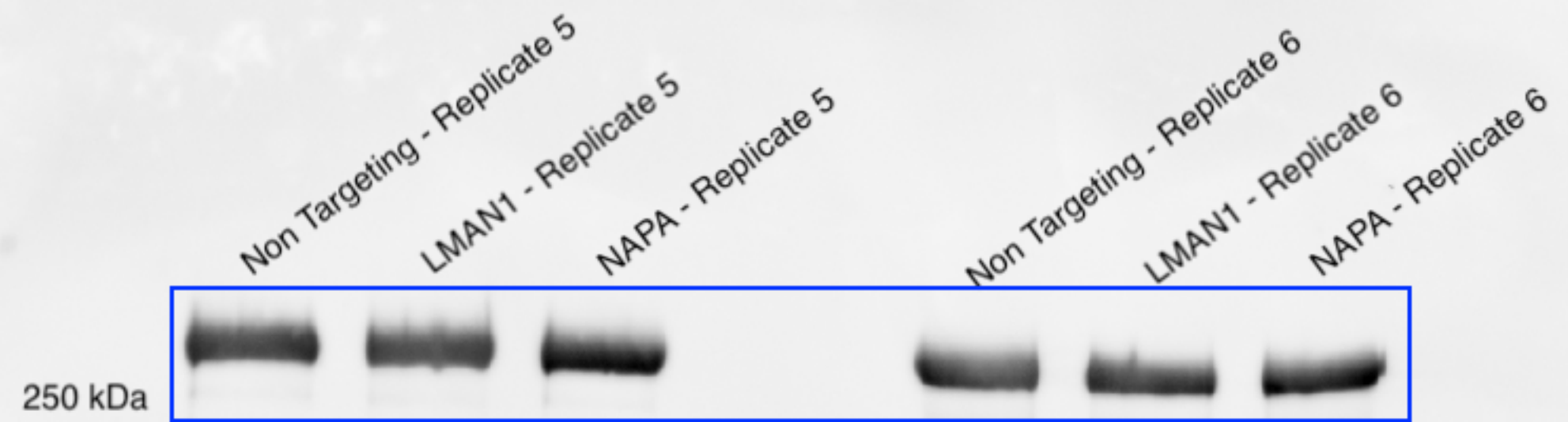

Supplement: Supplementary file 14 — Source data Fig. 5 [file 44320_2024_58_MOESM14_ESM.zip › Figure 5/5A/Fig 5A - Media - M2 [FLAG] (StarBright B700) - Replicates 5&6.pdf]

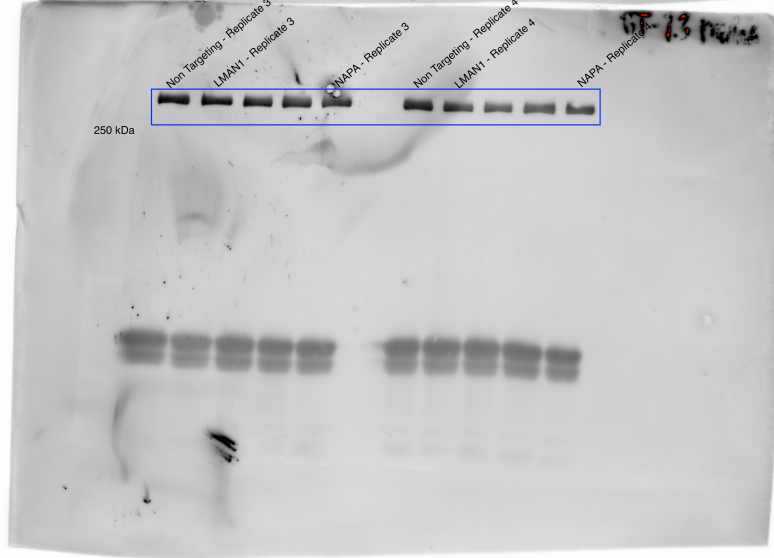

Supplement: Supplementary file 14 — Source data Fig. 5 [file 44320_2024_58_MOESM14_ESM.zip › Figure 5/5A/Fig5A - Media - M2 [FLAG] (StarBright B700) - Replicates 3&4.pdf]

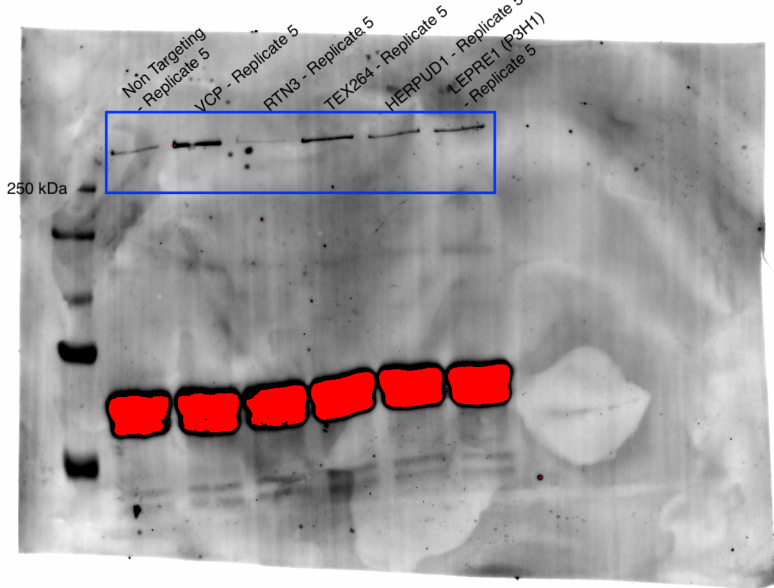

Supplement: Supplementary file 14 — Source data Fig. 5 [file 44320_2024_58_MOESM14_ESM.zip › Figure 5/5C/Fig 5C - Media - M2 [FLAG] (Starbright B700) - Replicate 5.pdf]

250 kDa

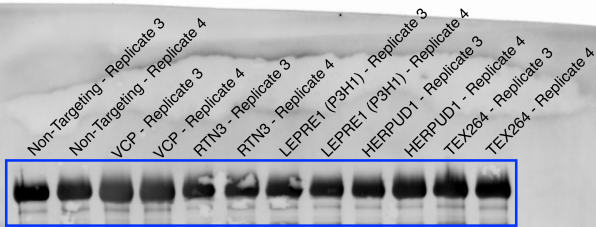

Supplement: Supplementary file 14 — Source data Fig. 5 [file 44320_2024_58_MOESM14_ESM.zip › Figure 5/5C/Fig 5C - Lysate - M2 [FLAG] (StarBright B700) - Replicates 3&4.pdf]

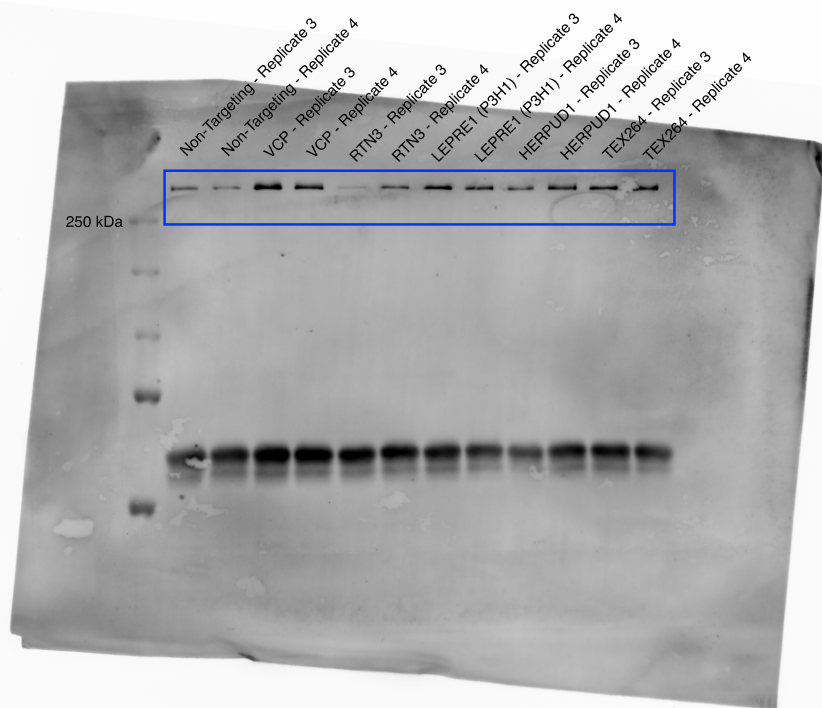

Supplement: Supplementary file 14 — Source data Fig. 5 [file 44320_2024_58_MOESM14_ESM.zip › Figure 5/5C/Fig 5C - Media - M2 [FLAG] (StarBright B700) - Replicates 3&4.pdf]

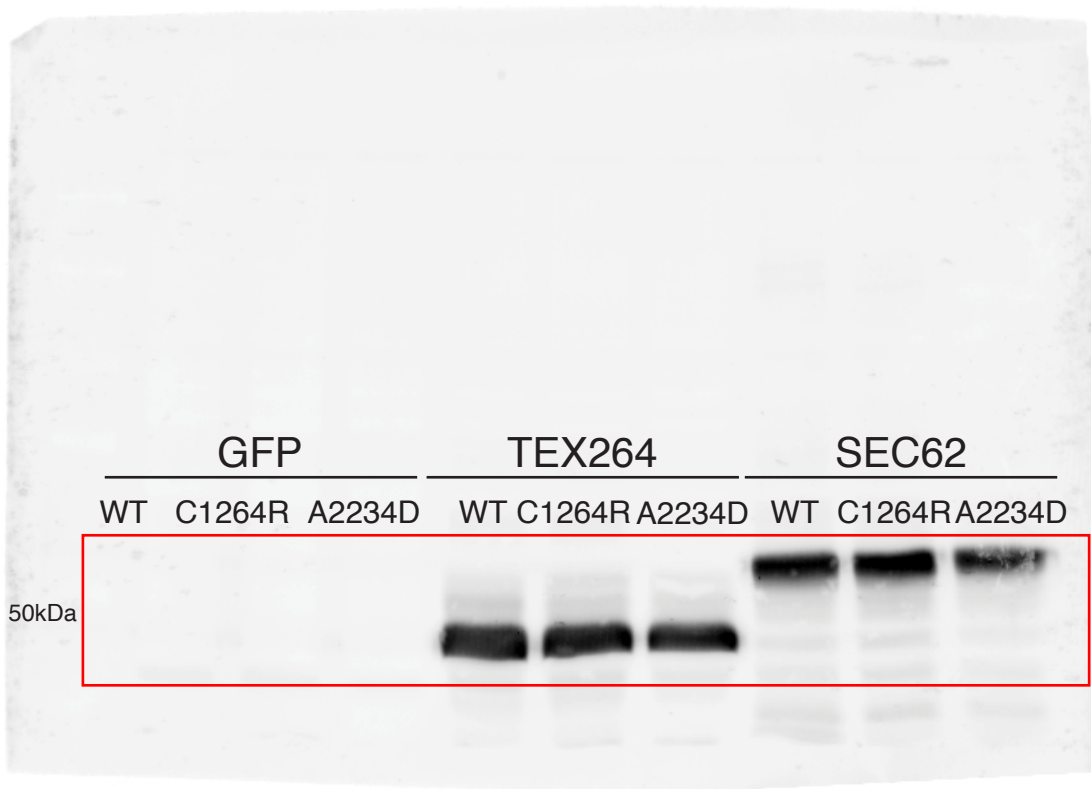

Supplement: Supplementary file 15 — Source data Fig. 6 [file 44320_2024_58_MOESM15_ESM.zip › Figure 6/6C/Fig 6C western-input-flag.pdf]

GFP

TEX264

SEC62

WT C1264R A2234D

WT C1264R A2234D

WT C1264R A2234D

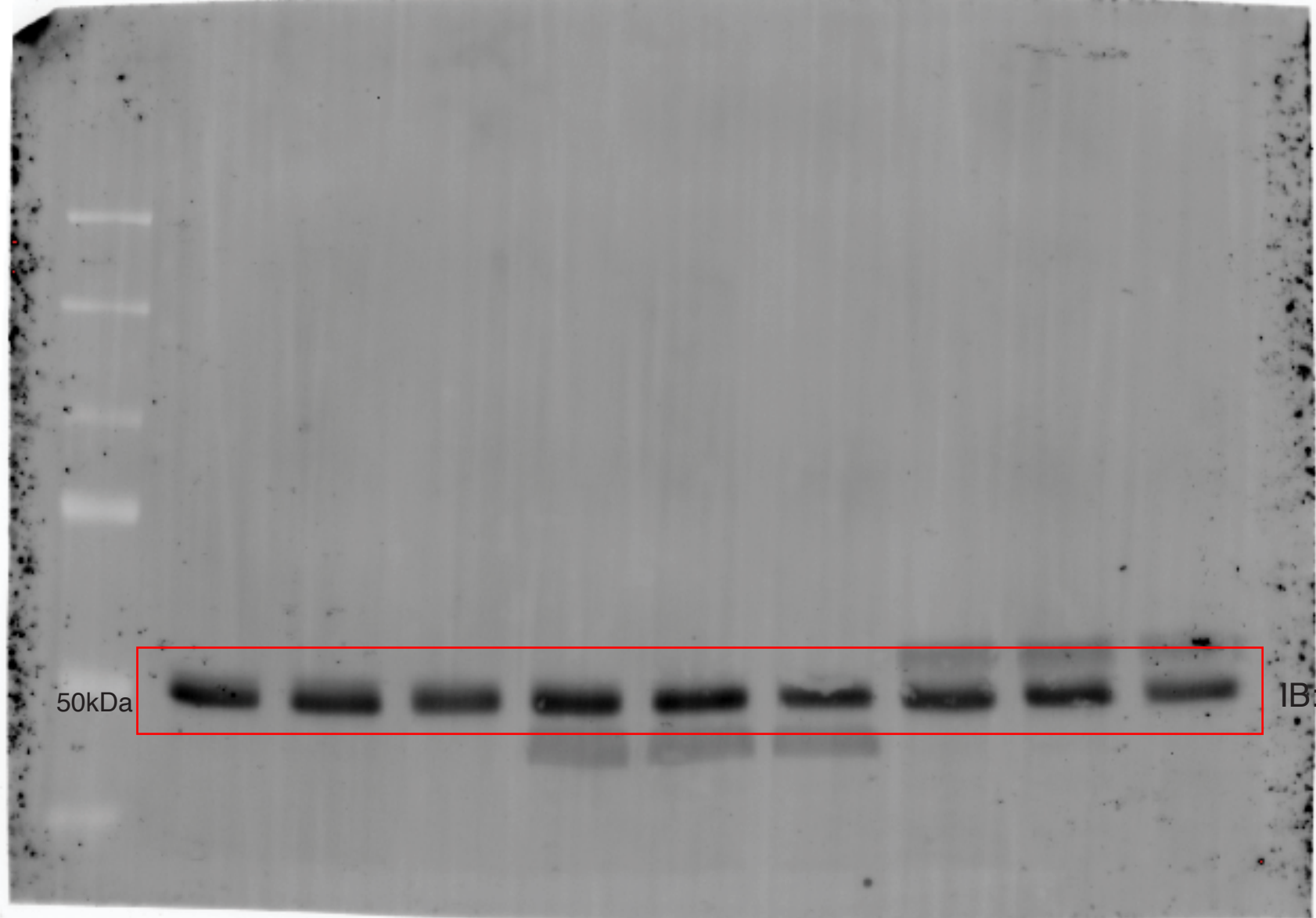

50kDa

IB: Tubulin

Supplement: Supplementary file 15 — Source data Fig. 6 [file 44320_2024_58_MOESM15_ESM.zip › Figure 6/6C/Fig 6C western-input-tubulin.pdf]

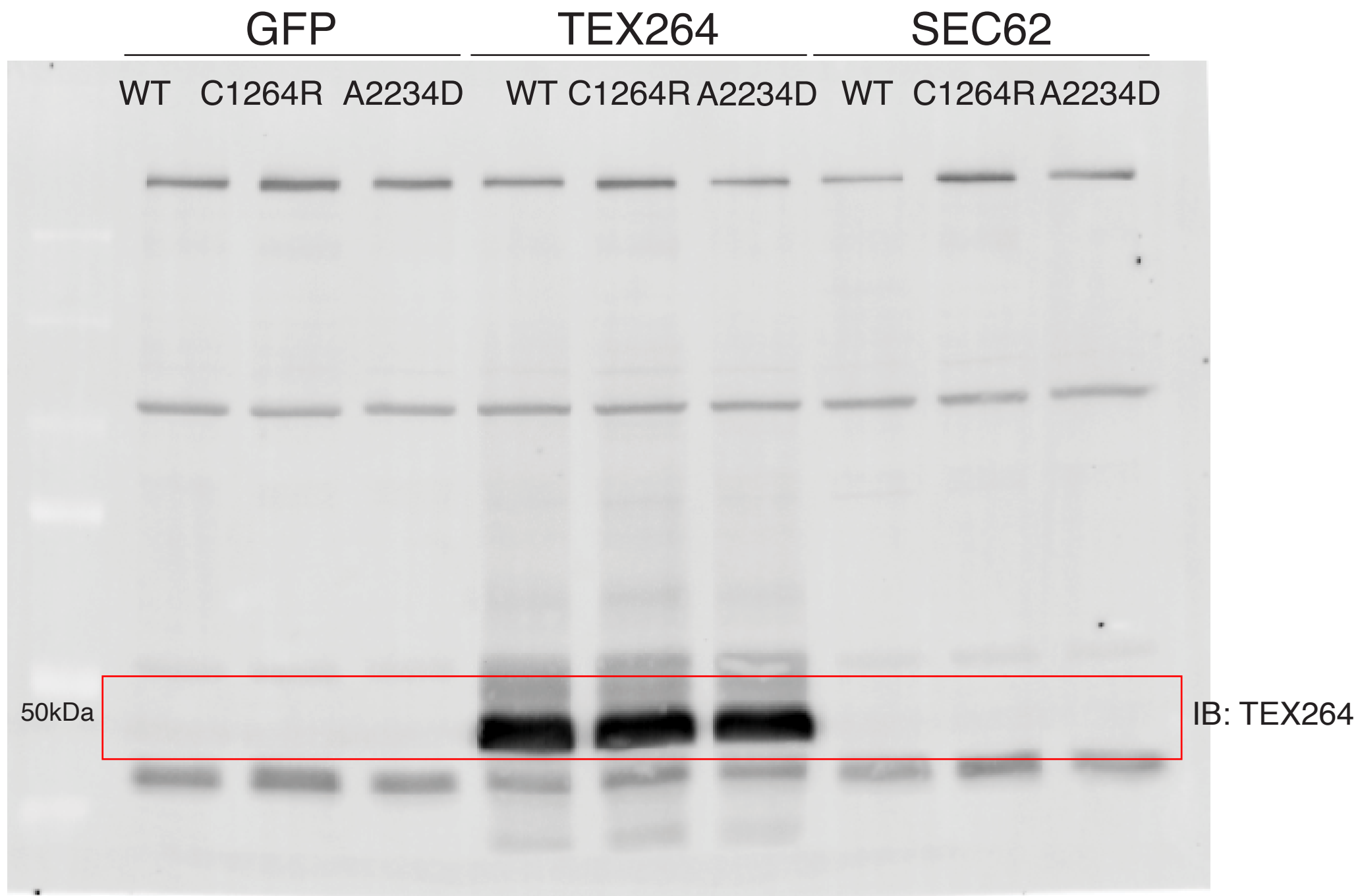

Supplement: Supplementary file 15 — Source data Fig. 6 [file 44320_2024_58_MOESM15_ESM.zip › Figure 6/6C/Fig 6C western-input-tex.pdf]

GFP

TEX264

SEC62

WT C1264R A2234D

WT C1264R A2234D

WT C1264R A2234D

250kDa

IB: Tg

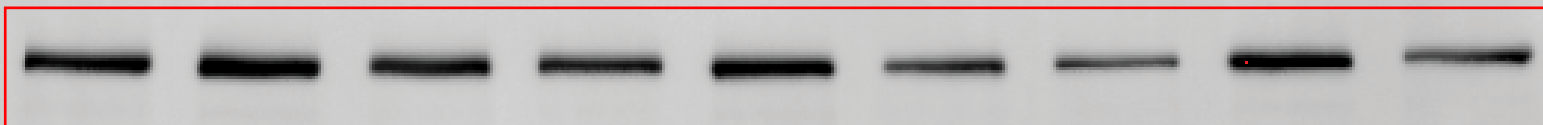

Supplement: Supplementary file 15 — Source data Fig. 6 [file 44320_2024_58_MOESM15_ESM.zip › Figure 6/6C/Fig 6C western-input-tg.pdf]

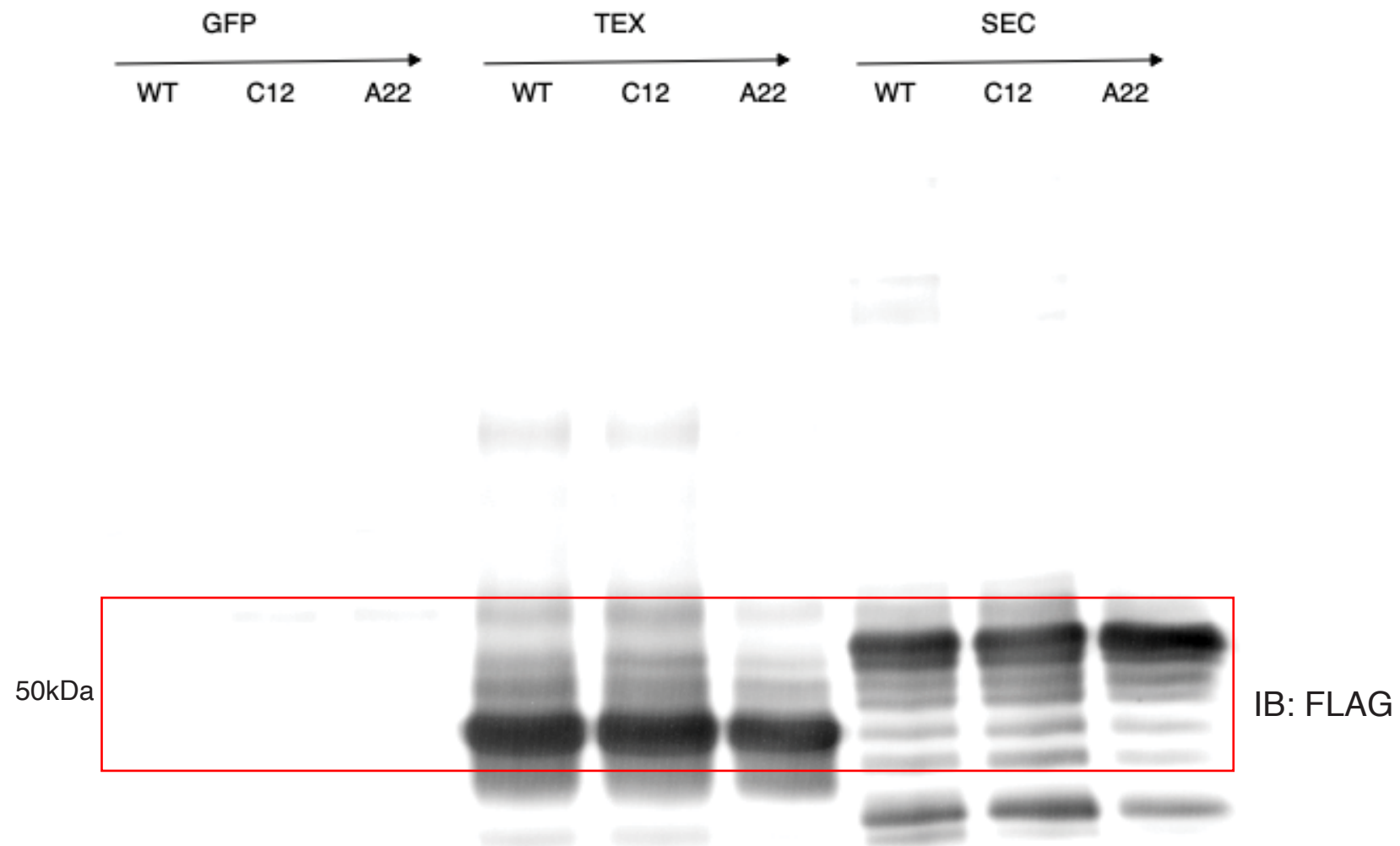

Supplement: Supplementary file 15 — Source data Fig. 6 [file 44320_2024_58_MOESM15_ESM.zip › Figure 6/6C/Fig 6C western-elution-flag.pdf]

GFP                      TEX                      SEC  
WT   C12   A22      WT   C12   A22      WT   C12   A22

50kDa

IB: TEX264

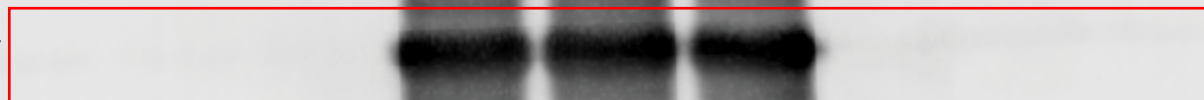

Supplement: Supplementary file 15 — Source data Fig. 6 [file 44320_2024_58_MOESM15_ESM.zip › Figure 6/6C/Fig 6C western-elution-tex.pdf]

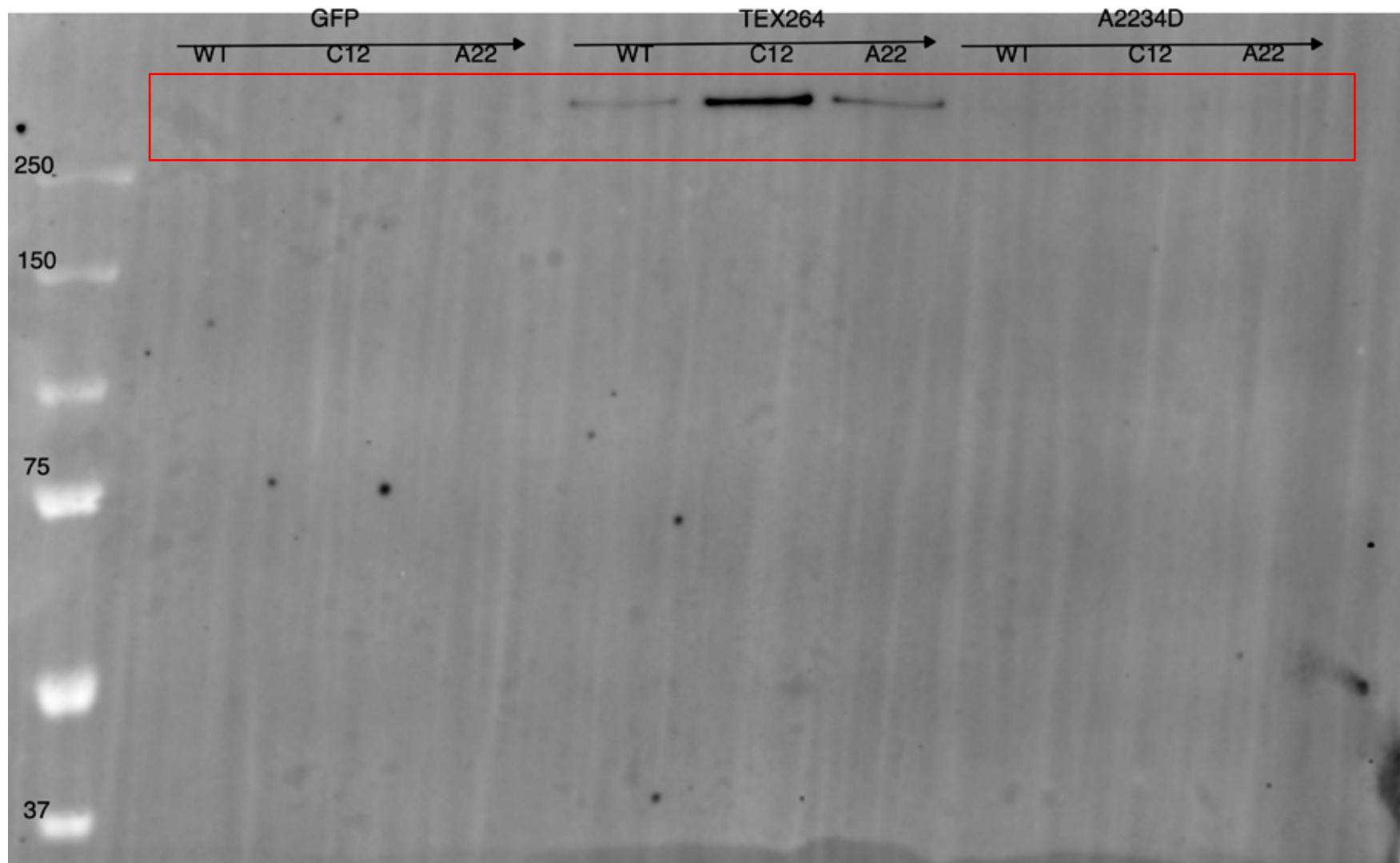

IB: Tg

Supplement: Supplementary file 15 — Source data Fig. 6 [file 44320_2024_58_MOESM15_ESM.zip › Figure 6/6C/Fig 6C western-elution-tg.pdf]

65-240

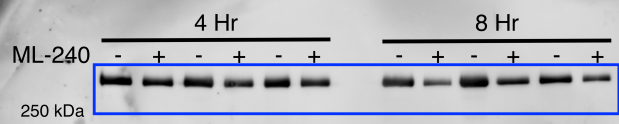

Supplement: Supplementary file 16 — Source data Fig. 7 [file 44320_2024_58_MOESM16_ESM.zip › Figure 7/7C/Fig 7C - Lysate - M2 [FLAG] (Starbright B700) - Replicates 1-3.pdf]

05-18-2014

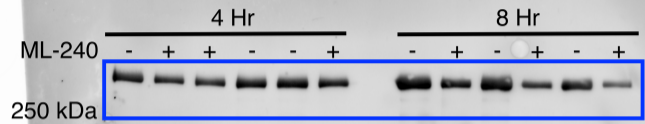

Supplement: Supplementary file 16 — Source data Fig. 7 [file 44320_2024_58_MOESM16_ESM.zip › Figure 7/7C/Fig 7C - Media - M2 [FLAG] (Starbright B700) - Replicates 1-3.pdf]

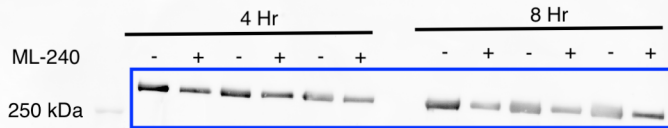

Supplement: Supplementary file 16 — Source data Fig. 7 [file 44320_2024_58_MOESM16_ESM.zip › Figure 7/7C/Fig 7C - Media - M2 [FLAG] (Starbright B700) - Replicates 4-6 .pdf]

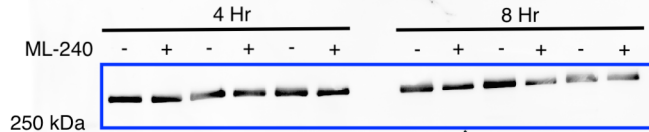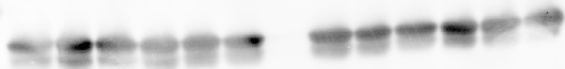

Supplement: Supplementary file 16 — Source data Fig. 7 [file 44320_2024_58_MOESM16_ESM.zip › Figure 7/7C/Fig 7C - Lysate - M2 [FLAG] (Starbright B700) - Replicates 4-6 .pdf]

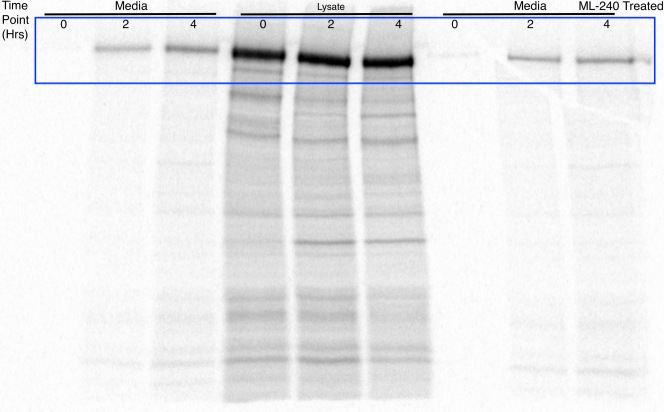

Supplement: Supplementary file 16 — Source data Fig. 7 [file 44320_2024_58_MOESM16_ESM.zip › Figure 7/7E/Fig 7E - Gel H.pdf]

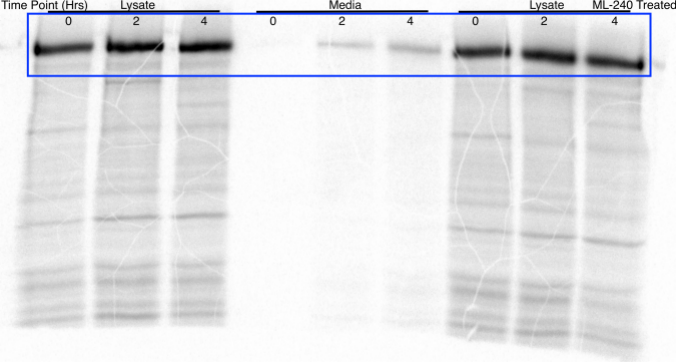

Supplement: Supplementary file 16 — Source data Fig. 7 [file 44320_2024_58_MOESM16_ESM.zip › Figure 7/7E/Fig 7E - Gel C.pdf]

Time Point (Hrs) Media Lysate Media DMSO Treated

0 2 4 0 2 4 0 2 4

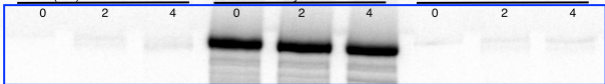

Supplement: Supplementary file 16 — Source data Fig. 7 [file 44320_2024_58_MOESM16_ESM.zip › Figure 7/7E/Fig 7E - Gel B.pdf]

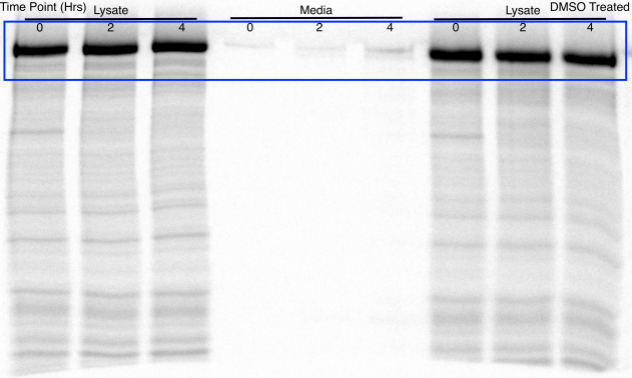

Supplement: Supplementary file 16 — Source data Fig. 7 [file 44320_2024_58_MOESM16_ESM.zip › Figure 7/7E/Fig 7E - Gel A.pdf]

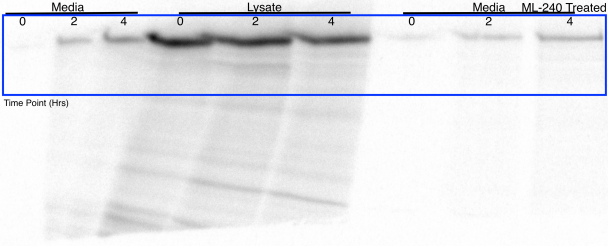

Supplement: Supplementary file 16 — Source data Fig. 7 [file 44320_2024_58_MOESM16_ESM.zip › Figure 7/7E/Fig 7E - Gel D.pdf]

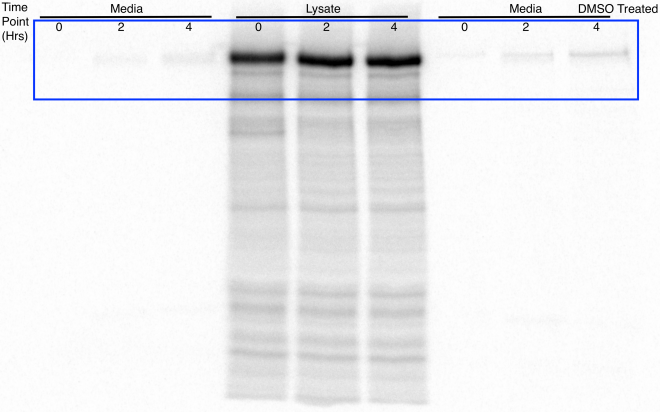

Supplement: Supplementary file 16 — Source data Fig. 7 [file 44320_2024_58_MOESM16_ESM.zip › Figure 7/7E/Fig 7E - Gel F.pdf]

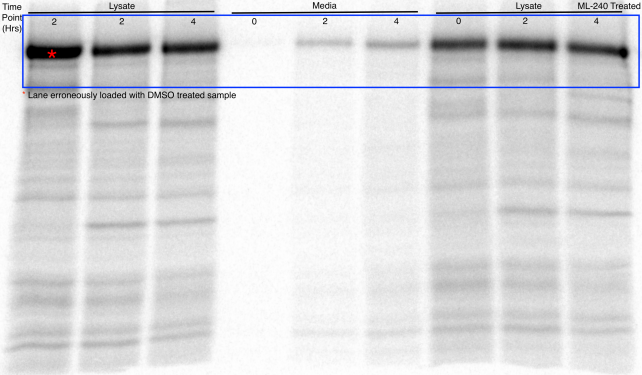

Supplement: Supplementary file 16 — Source data Fig. 7 [file 44320_2024_58_MOESM16_ESM.zip › Figure 7/7E/Fig 7E - Gel G.pdf]

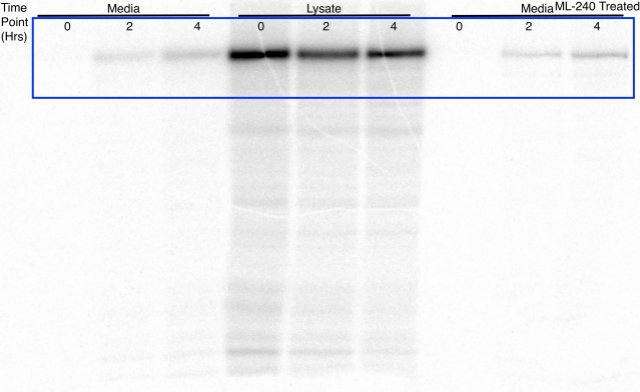

Supplement: Supplementary file 16 — Source data Fig. 7 [file 44320_2024_58_MOESM16_ESM.zip › Figure 7/7F/Fig 7F - Gel H.pdf]

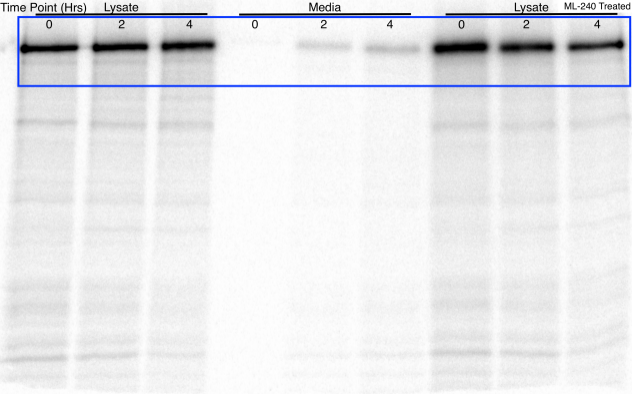

Supplement: Supplementary file 16 — Source data Fig. 7 [file 44320_2024_58_MOESM16_ESM.zip › Figure 7/7F/Fig 7F - Gel G.pdf]

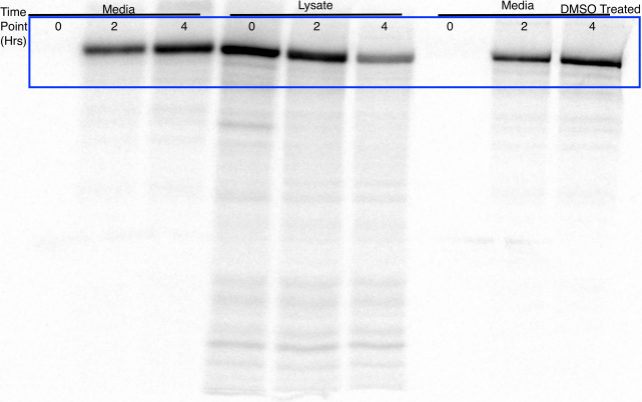

Supplement: Supplementary file 16 — Source data Fig. 7 [file 44320_2024_58_MOESM16_ESM.zip › Figure 7/7F/Fig 7F - Gel F.pdf]

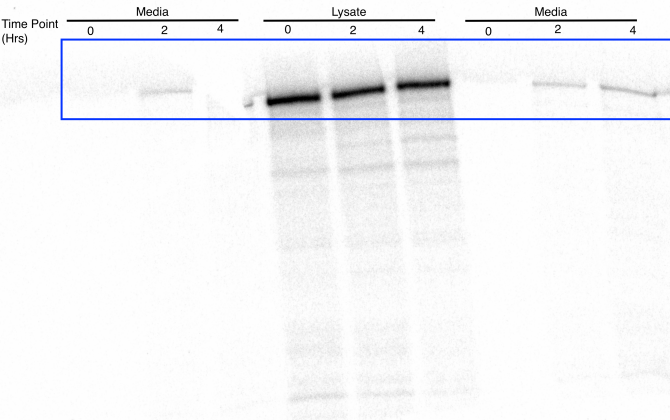

Supplement: Supplementary file 16 — Source data Fig. 7 [file 44320_2024_58_MOESM16_ESM.zip › Figure 7/7F/Fig 7F - Gel D.pdf]

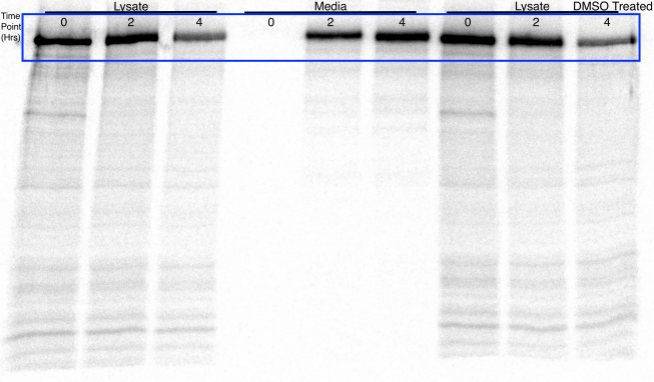

Supplement: Supplementary file 16 — Source data Fig. 7 [file 44320_2024_58_MOESM16_ESM.zip › Figure 7/7F/Fig 7F - Gel E.pdf]

Time Point (Hrs)

| Lysate |   |   | Media |   |   | Lysate |   |   |
|--------|---|---|-------|---|---|--------|---|---|
| 0      | 2 | 4 | 0     | 2 | 4 | 0      | 2 | 4 |

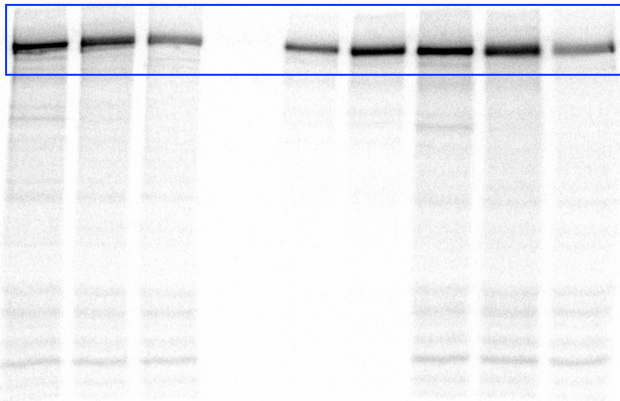

Supplement: Supplementary file 16 — Source data Fig. 7 [file 44320_2024_58_MOESM16_ESM.zip › Figure 7/7F/Fig 7F - Gel A.pdf]

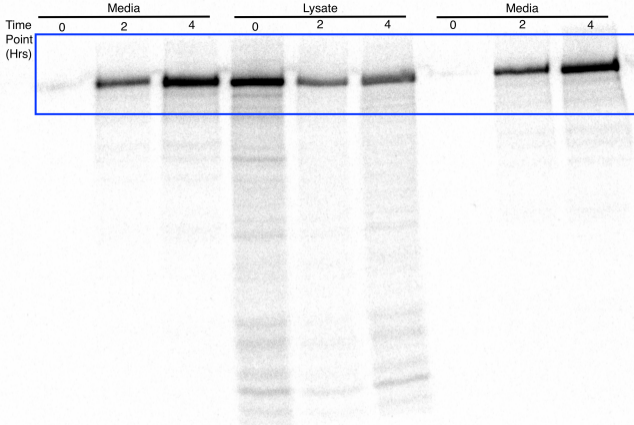

Supplement: Supplementary file 16 — Source data Fig. 7 [file 44320_2024_58_MOESM16_ESM.zip › Figure 7/7F/Fig 7F - Gel B.pdf]

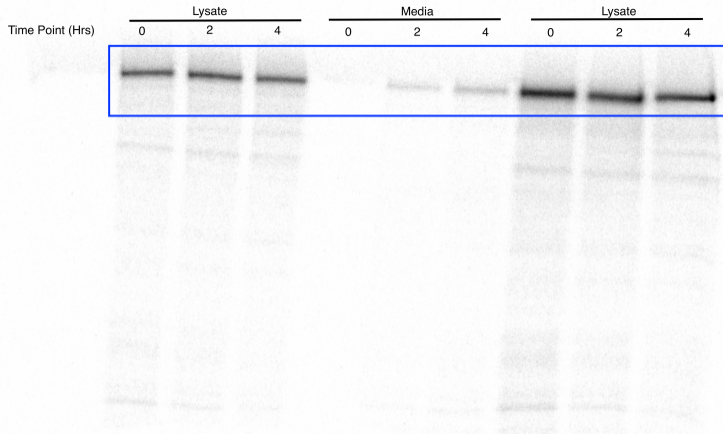

Supplement: Supplementary file 16 — Source data Fig. 7 [file 44320_2024_58_MOESM16_ESM.zip › Figure 7/7F/Fig 7F - Gel C.pdf]

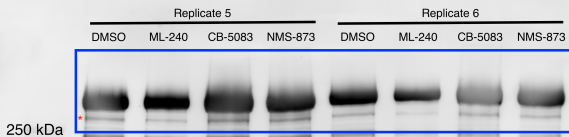

Supplement: Supplementary file 16 — Source data Fig. 7 [file 44320_2024_58_MOESM16_ESM.zip › Figure 7/7A/Fig 7A - Lysate - M2 [FLAG] (StarBright B700) - Replicates 5&6.pdf]

250 kDa

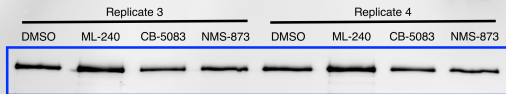

Supplement: Supplementary file 16 — Source data Fig. 7 [file 44320_2024_58_MOESM16_ESM.zip › Figure 7/7A/Fig 7A - Media - M2 [FLAG] (StarBright B700) - Replicates 3&4 .pdf]

0.45 L NDA

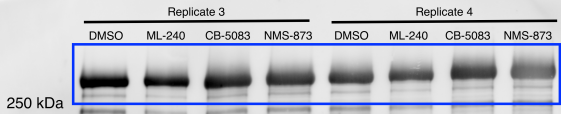

Supplement: Supplementary file 16 — Source data Fig. 7 [file 44320_2024_58_MOESM16_ESM.zip › Figure 7/7A/Fig 7A - Lysate - M2 [FLAG] (StarBright B700) - Replicates 3&4.pdf]

85-15 v01102

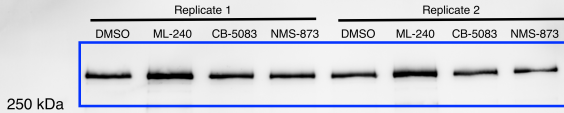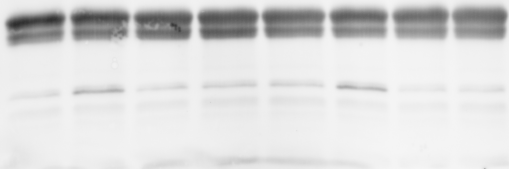

Supplement: Supplementary file 16 — Source data Fig. 7 [file 44320_2024_58_MOESM16_ESM.zip › Figure 7/7A/Fig 7A - Media - M2 [FLAG] (StarBright B700) - Replicates 1&2 .pdf]

250 kDa

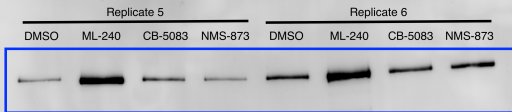

Supplement: Supplementary file 16 — Source data Fig. 7 [file 44320_2024_58_MOESM16_ESM.zip › Figure 7/7A/Fig 7A - Media - M2 [FLAG] (StarBright B700) - Replicates 5&6.pdf]
