# Supplementary material for: Time-resolved interactome profiling deconvolutes secretory protein quality control dynamics
Source: Mol Syst Biol. 2024 Aug 5;20(9):1049–75. doi: 10.1038/s44320-024-00058-1 (PMC11369088; doi:10.1038/s44320-024-00058-1)
Supplement: Supplementary file 14 — Source data Fig. 5 [file 44320_2024_58_MOESM14_ESM.zip › Figure 5/5A/Fig5A - Lysate - M2 [FLAG] (StarBright B700) - Replicates 3&4.pdf]

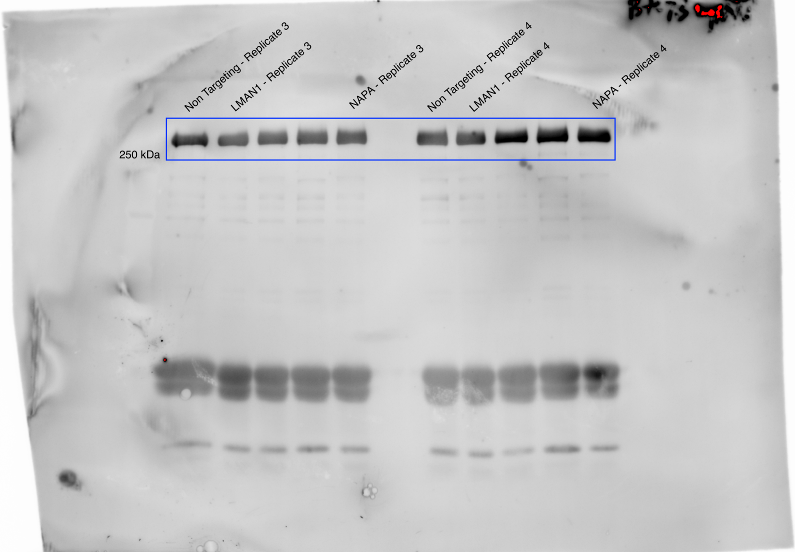
Non Targeting - Replicate 3
LMAN1 - Replicate 3
NAPA - Replicate 3
Non Targeting - Replicate 4
LMAN1 - Replicate 4
NAPA - Replicate 4
250 kDa
