# Supplementary material for: Time-resolved interactome profiling deconvolutes secretory protein quality control dynamics
Source: Mol Syst Biol. 2024 Aug 5;20(9):1049–75. doi: 10.1038/s44320-024-00058-1 (PMC11369088; doi:10.1038/s44320-024-00058-1)
Supplement: Supplementary file 14 — Source data Fig. 5 [file 44320_2024_58_MOESM14_ESM.zip › Figure 5/5A/Fig 5A - Media - M2 [FLAg] (StarBright B700) - Replicates 1&2.pdf]

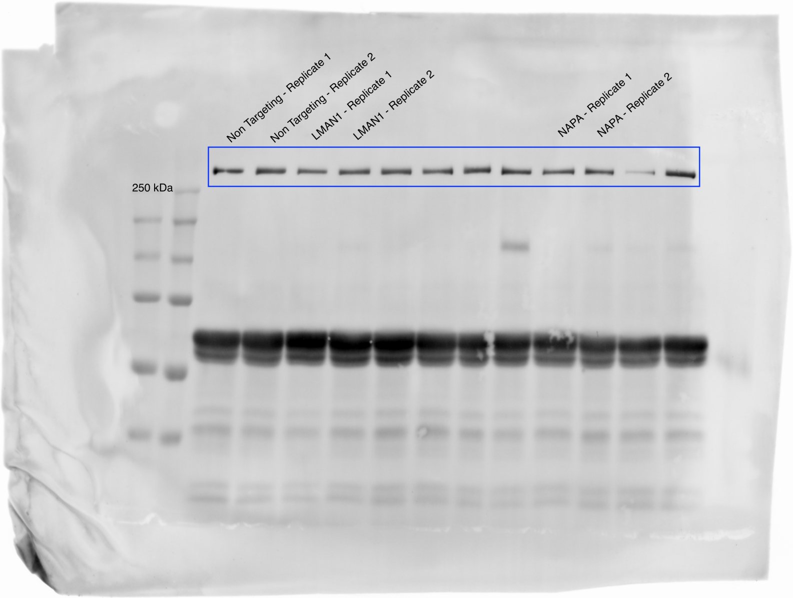

Non Targeting - Replicate 1
Non Targeting - Replicate 2
LMAN1 - Replicate 1
LMAN1 - Replicate 2
NAPA - Replicate 1
NAPA - Replicate 2
250 kDa
