# Supplementary material for: Time-resolved interactome profiling deconvolutes secretory protein quality control dynamics
Source: Mol Syst Biol. 2024 Aug 5;20(9):1049–75. doi: 10.1038/s44320-024-00058-1 (PMC11369088; doi:10.1038/s44320-024-00058-1)
Supplement: Supplementary file 16 — Source data Fig. 7 [file 44320_2024_58_MOESM16_ESM.zip › Figure 7/7E/Fig 7E - Gel E.pdf]

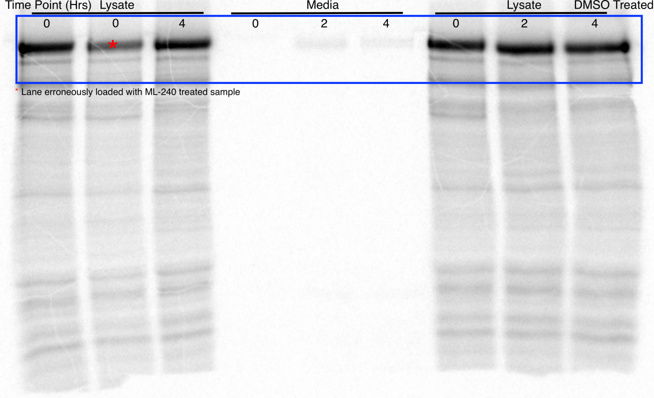
Time Point (Hrs)
Lysate
Media
Lysate
DMSO Treated
0
0
4
0
2
4
0
2
4
*
* Lane erroneously loaded with ML-240 treated sample
