# Supplementary material for: Time-resolved interactome profiling deconvolutes secretory protein quality control dynamics
Source: Mol Syst Biol. 2024 Aug 5;20(9):1049–75. doi: 10.1038/s44320-024-00058-1 (PMC11369088; doi:10.1038/s44320-024-00058-1)
Supplement: Supplementary file 17 — Expanded View Figures [file 44320_2024_58_MOESM17_ESM.pdf]

Expanded View Figures

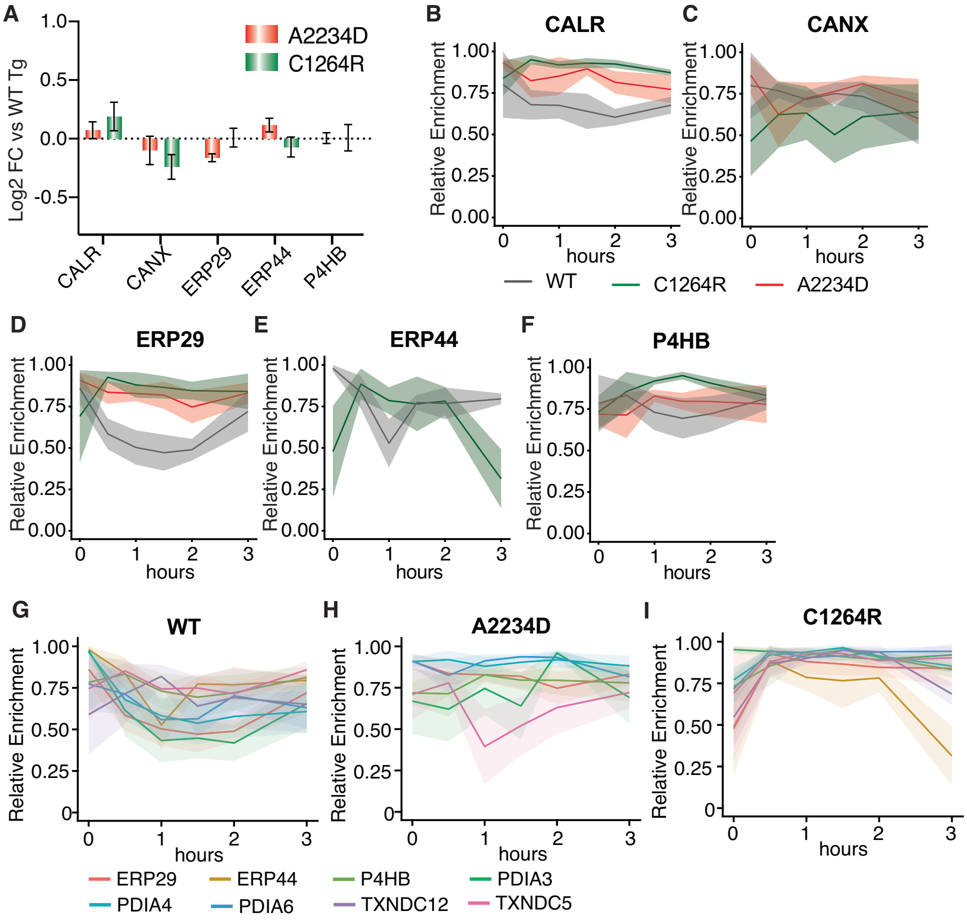

Figure EV1. TRIP data for individual interactors.

(**A**) Aggregate (steady-state) interactomics data comparing the enrichment of Tg interactors for mutant Tg to WT Tg (data from Wright et al, 2021). Interactions are mostly unchanged for mutant Tg relative to WT Tg. Data is represented as mean ± SEM ($N = 12$ biological replicates for C1264R, and 6 biological replicates for A2234D). (**B–F**) Plots comparing the relative enrichment of interactors CALR (**B**), CANX (**C**), ERP29 (**D**), ERP44 (**E**), and P4HB (**F**) throughout the TRIP time course for WT, A2234D, and C1264R Tg. TRIP data can resolve dynamic interaction changes for several mutant Tg interactors, while these changes are muted in the aggregate data. Solid line corresponds to mean and shading represents the SEM ($N = 5$ for WT Tg; $N = 6$ biological replicates for A2234D and C1264R Tg). (**G–I**) Plots comparing the relative enrichment of individual disulfide/redox-processing interactors throughout the TRIP time course for WT (**G**), A2234D (**H**), and C1234R (**I**). Individual protein disulfide isomerases exhibit distinct peak times when interactions reach maximum, thereby revealing an order to their engagement. For instance, PDIA3, PDIA4, PDIA6, and P4HB peak at 0 h for WT Tg, while TXNDC12 peaks later at 1 h. Moreover, the exact temporal sequence of PDI engagements is shifted for A2234D (**H**) and C1234R Tg (**I**). Solid line corresponds to mean and shading represents the SEM ($N = 5$ for WT Tg; $N = 6$ biological replicates for A2234D and C1264R Tg). Data available in Dataset EV4.

© The Author(s)

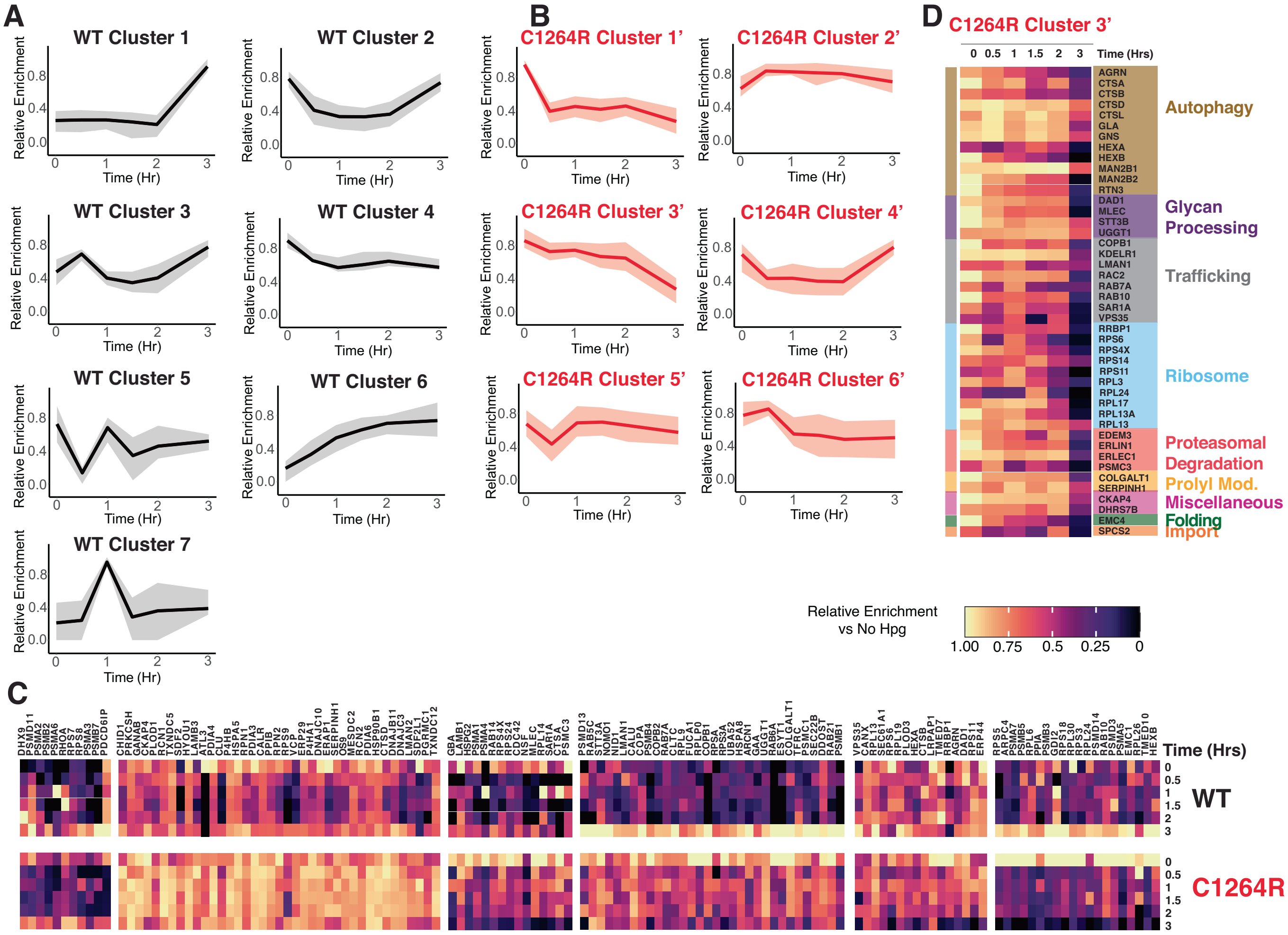

Figure EV2. Unbiased clustering of TRIP data.

(**A**, **B**) Unbiased k-means clustering of TRIP profiles for WT (**A**) and C1264R (**B**) to determine co-regulated groups of interactors. k-means clustering was carried out by using the k-means function from the tslearn python package with the data being normalized using the scaler mean variance function. This analysis resulted in 7 distinct clusters for WT and 6 clusters for C1264R. The line corresponds to the mean scaled log2 fold enrichment and the shading represents the 25–75 % quarter range within each cluster. (**C**) Heatmap showing unbiased k-means clustering of the combined WT and C1264R Tg TRIP profiles. Only interactors identified in both datasets were included. (**D**) Heatmap for interactors in C1264R Cluster 3 (from **B**), which displayed the strongest interactions at the initial 0 h timepoint. The scaled log2 fold change enrichment for individual interactors is shown, and the individual interactors are grouped by pathway. Several interactors related to autophagy (brown) and glycan processing (purple), including the glycoprotein folding sensor UGGT1, are present in this cluster.

© The Author(s)

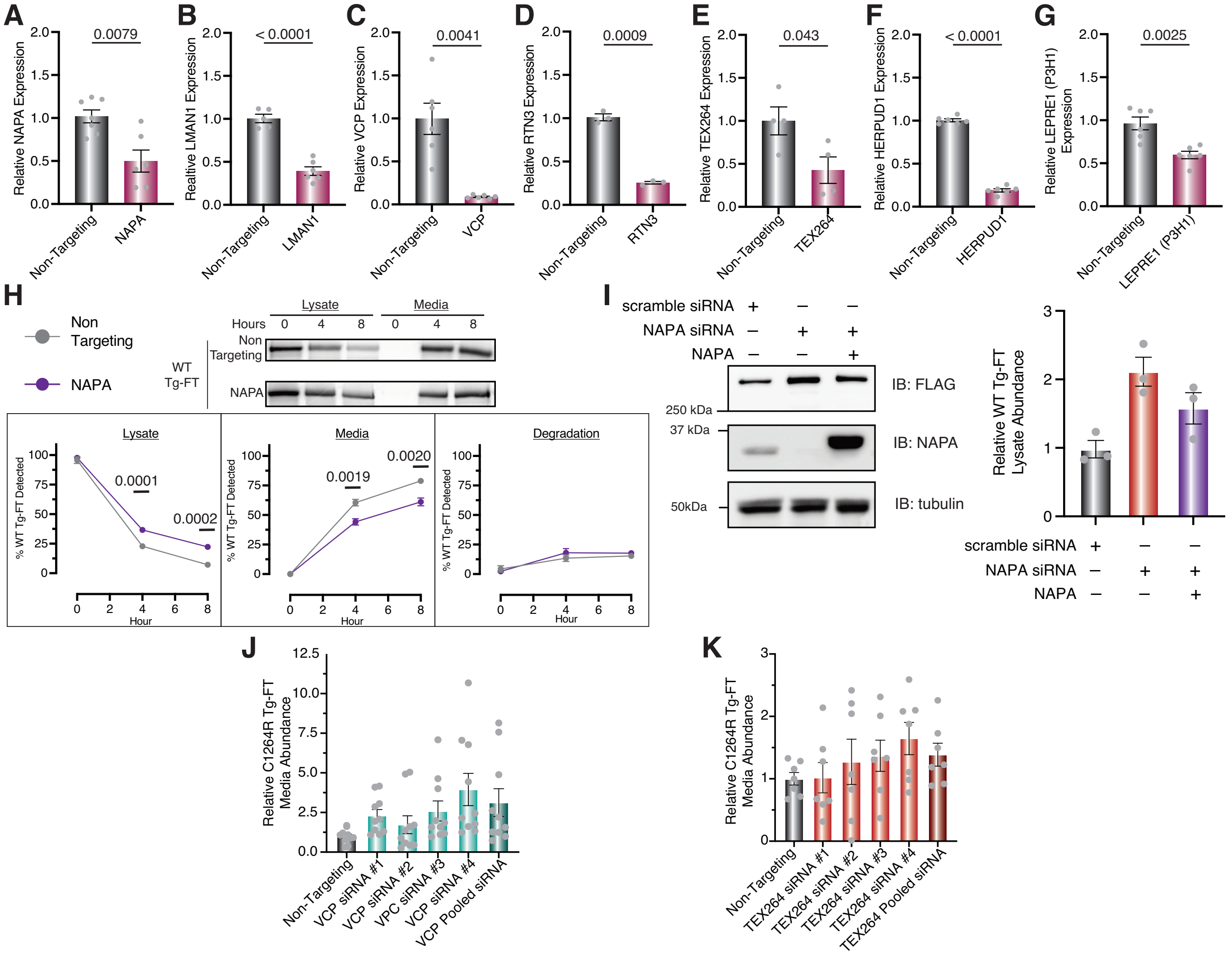

Figure EV3. Validation of siRNA screening hits in FRT cells.

(A–G) Relative expression of knockdown targets in engineered WT Tg-FT FRT cells with and without siRNA silencing measured by qRT-PCR. Data was first normalized to a GAPDH loading control followed by normalization to median expression of non-targeting transfected samples and represented as mean ± SEM. **(A)** NAPA (α-SNAP), **(B)** LMAN1, **(C)** VCP, **(D)** RTN3, **(E)** TEX264, **(F)** HERPUD1, **(G)** LEPRE11 (P3H1). Statistical testing was performed using an unpaired Student's *t* test with Welch's correction with *P* values as indicated. *N* = 3–7 biological replicates as shown. Primers for detection are described in Dataset EV7. **(H)** Pulse-chase analysis of WT Tg-FT in FRT cells with NAPA (α-SNAP) siRNA knockdown. Approximately 36 h after transfection with 25 nM siRNAs cells were pulse-labeled with EasyTag ^{35}S Protein Labeling Mix (Perkin Elmer, NEG772007MC) for 30 min and chased for 8 h, collecting samples at 0-, 4-, and 8-h time points. Data is normalized to timepoint of maximum Tg recovery and represented as mean ± SEM. % Degradation is defined as $\left[1-\left(Tg_t^{lysate}+Tg_t^{media}\right)\right] x\,100$. Where Tg_t^{lysate} is the fraction of Tg-FT detected in the lysate at a given timepoint *n*, and Tg_t^{media} is the fraction of Tg-FT detected in the media at a given timepoint n. Statistical testing performed using an unpaired Student's *t* test with Welch's correction with *P* values as indicated. *N* = 6 biological replicates. **(I)** Complementation of NAPA knockdown partially reverses WT-Tg retention. FRT cells stably expressing WT Tg were cotransfected with NAPA siRNA and siRNA resistant NAPA expression plasmid. Cells were harvested 40 h post transfection and lysates were analyzed by Western blot to monitor changes in WT-Tg amounts. Quantification (mean ± SEM) is shown on the right (*N* = 3 biological replicates). **(J, K)** Individual siRNA knockdown of VCP **(J)** and TEX265 **(K)** in C1264R Tg-FRT cells to confirm the increase in Tg secretion. Cells were transfected with 25 nM siRNAs for 36 h, media exchanged and conditioned for 8 h, Tg-FT was immunoprecipitated from media samples, and Tg-FT amounts were analyzed via immunoblotting. Multiple individual siRNAs recapitulated the increase in C1264R secretion. Data is represented as mean ± SEM for *N* = 10 **(J)** or 7 **(K)** biological replicates.

 © The Author(s)

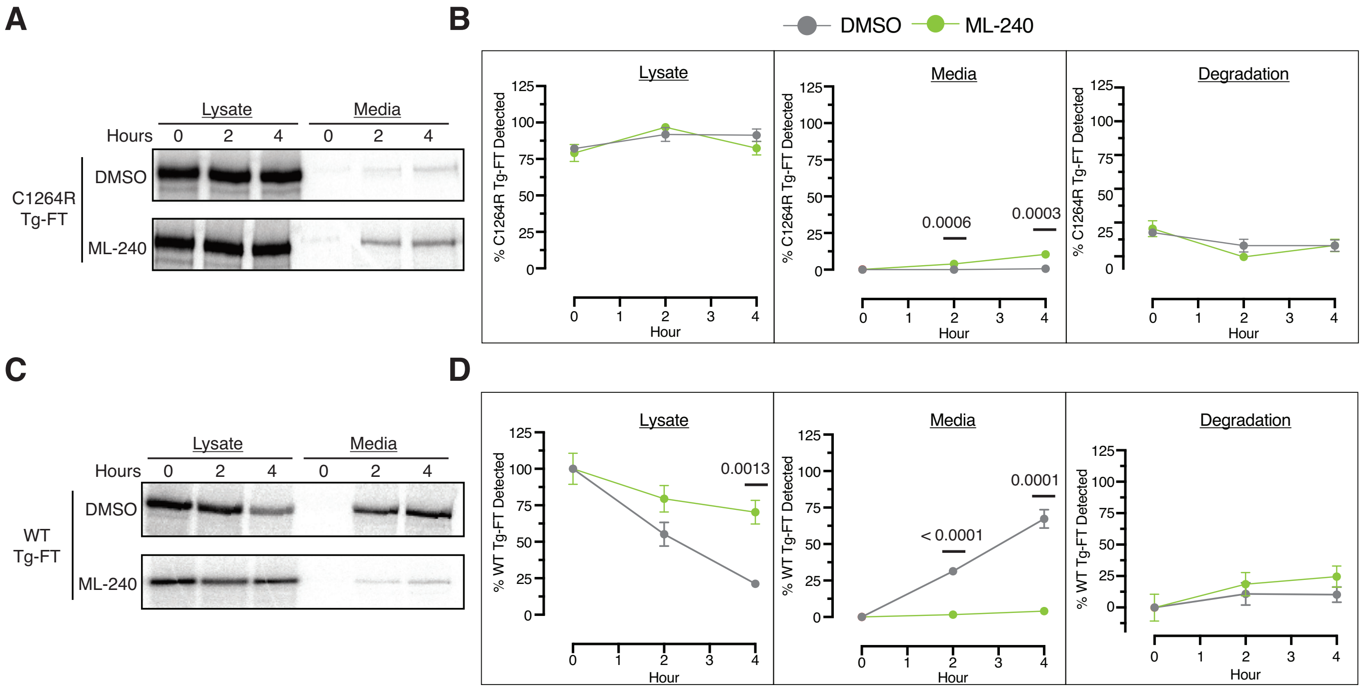

Figure EV4. Pulse-chase analysis of WT and C1264R Tg with pharmacological VCP inhibition.

Autoradiographs and quantifications of pulse-chase analysis of C1264R Tg-FT (**A**, **B**) and WT Tg-FT (**C**, **D**) in FRT cells with ML-240 treatment. Cells were pre-treated with ML-240 or DMSO for 15 min prior to pulse labeling with EasyTag ^{35}S Protein Labeling Mix (Perkin Elmer, NEG772007MC) for 30 min and chased for 4 h with DMSO or ML-240 treatment, collecting samples at 0-, 2-, and 4-h time points. Autoradiographs from a representative experiment are shown in (**A**, **C**). Quantification is shown in (**C**, **D**). Data is normalized to the timepoint of maximum Tg recovery (C1264R) or 0 h (WT) and represented as mean ± SEM. Statistical testing was performed using an unpaired Student's *t* test with Welch's correction with *P* values as indicated. *N* = 5–6 biological replicates as shown.

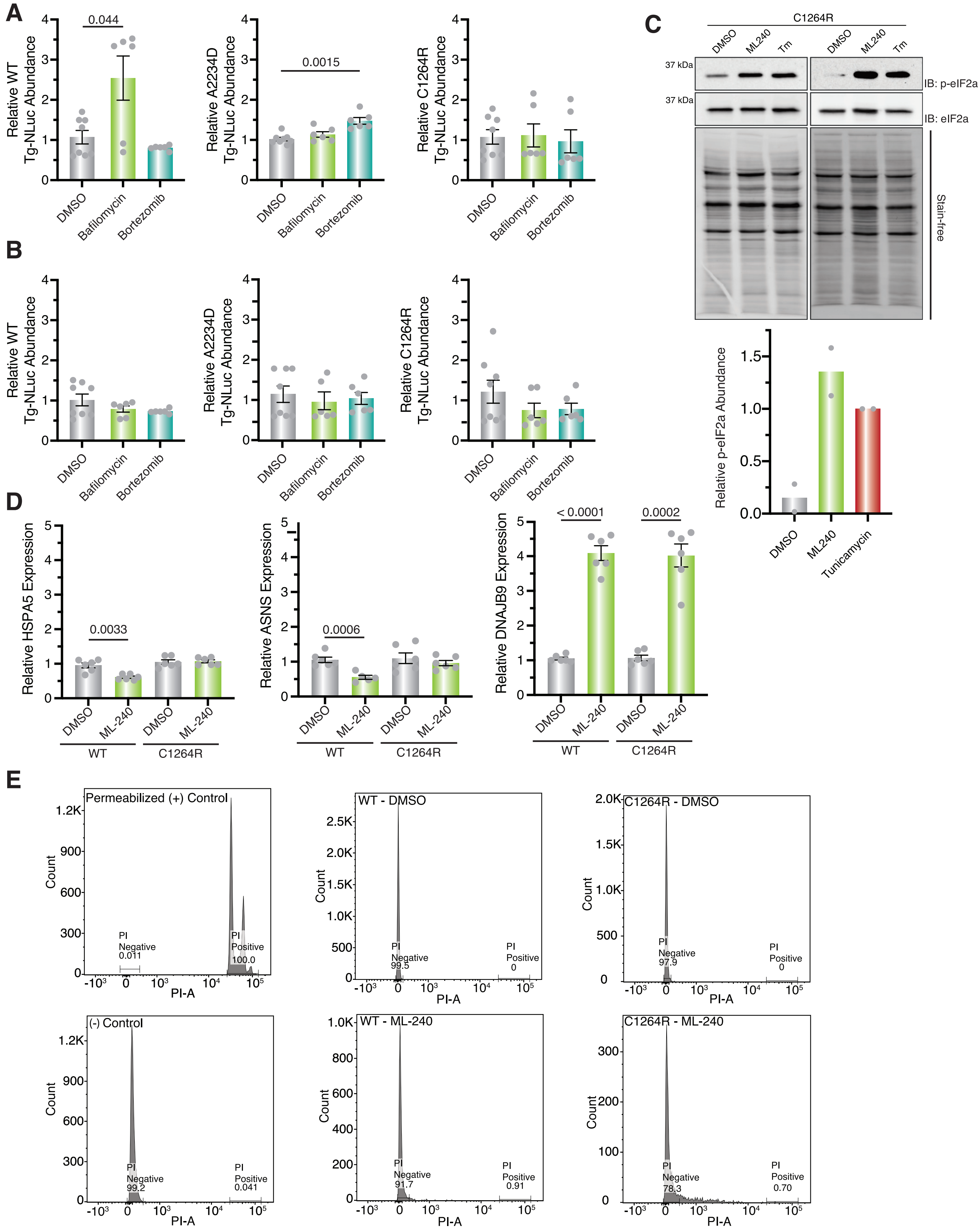
A
Relative WT Tg-NLuc Abundance
0.044
Relative A2234D Tg-NLuc Abundance
0.0015
Relative C1264R Tg-NLuc Abundance
DMSO
Bafilomycin
Bortezomib
B
Relative WT Tg-NLuc Abundance
Relative A2234D Tg-NLuc Abundance
Relative C1264R Tg-NLuc Abundance
C
C1264R
DMSO
ML240
Tm
37 kDa
IB: p-eIF2a
IB: eIF2a
Stain-free
Relative p-eIF2a Abundance
Tunicamycin
D
Relative HSPA5 Expression
0.0033
Relative ASNS Expression
0.0006
Relative DNAJB9 Expression
< 0.0001
0.0002
DMSO
ML-240
WT
C1264R
E
Permeabilized (+) Control
PI Negative 0.011
PI Positive 100.0
WT - DMSO
PI Negative 99.5
PI Positive 0
C1264R - DMSO
PI Negative 97.9
PI Positive 0
(-) Control
PI Negative 99.2
PI Positive 0.041
WT - ML-240
PI Negative 91.7
PI Positive 0.91
C1264R - ML-240
PI Negative 78.3
PI Positive 0.70
Count
PI-A

© The Author(s)

Figure EV5. TRIP of C1264R Tg-FT FRT cells with pharmacological VCP inhibition.

(**A**, **B**) Effect of pharmacologic inhibition of protein degradation in Tg retention and secretion. HEK293T cells stably expressed Tg-NanoLuc variants were treated with proteasomal degradation inhibitor Bortezomib (10 μM) or lysosomal inhibitor Bafilomycin A1 (10 μM) for 8 h. Prior to treatment, the media was exchanged to condition secreted Tg. Tg lysate amounts (**A**) or media amounts (**B**) were then measured by the nano-glo luciferase assay system. Data is normalized to DMSO condition and represented as mean ± SEM. Statistical testing was performed using an unpaired Student's *t* test with Welch's correction with *P* values as indicated. $N = 6$–8 biological replicates as shown. (**C**, **D**) Assessment of UPR upregulation with ML-240 treatment. (**C**) Western blot analysis to assess phospho-eIF2α levels in of C1264R Tg-FT FRT cells treated with VCP inhibitor ML-240 (10 μM), tunicamycin (1 μg/mL), or vehicle (0.1% DMSO) for 2 h. Lysate samples were analyzed via immunoblotting. Data is normalized to the mean C1264R Tg-FT abundance of tunicamycin-treated samples. Data represented as mean ± SEM from $N = 2$ biological replicates. (**D**) Activation of UPR markers monitored via qPCR in C1264R Tg-FT FRT cells treated with ML-240 (10 μM) for 3 h. HSPA5 and ASNS expression remained unchanged in C2164R Tg-FT FRT cells but led to a significant decrease in WT Tg-FT FRT cells. Only DNAJB9 showed a significant increase in transcript levels for both C1264R and WT Tg-FT FRT cells. This suggest that ML-240 dependent rescue of C1264R Tg is not due to global remodeling of the ER proteostasis network via UPR activation. Data was first normalized to a GAPDH loading control followed by normalization to median expression of DMSO-treated samples and represented as mean ± SEM. Statistical testing performed using an unpaired Student's *t* test with Welch's correction with *P* values as indicated. $N = 6$ biological replicates. Primers for detection are described in Dataset EV7. (**E**) Viability analysis using Propidium iodide control samples, WT & C164R Tg-FT FRT cells with ML-240 treatment. Cells were treated with DMSO or ML-240 (10 μM) for 4 h, harvested, and stained with propidium iodide (1 μg/mL). FRT cells permeabilized with 0.2% Triton were used as a positive staining control. Unstained, non-permeabilized FRT cells were used as a negative staining control.

© The Author(s)
