# Supplementary material for: Time-resolved interactome profiling deconvolutes secretory protein quality control dynamics
Source: Mol Syst Biol. 2024 Aug 5;20(9):1049–75. doi: 10.1038/s44320-024-00058-1 (PMC11369088; doi:10.1038/s44320-024-00058-1)
Supplement: Supplementary file 16 — Source data Fig. 7 [file Fig 7A - Lysate - M2 [FLAG] (StarBright B700) - Replicates 1&2 .pdf › Figure 7/7A/Fig 7A - Lysate - M2 [FLAG] (StarBright B700) - Replicates 1&2 .pdf]

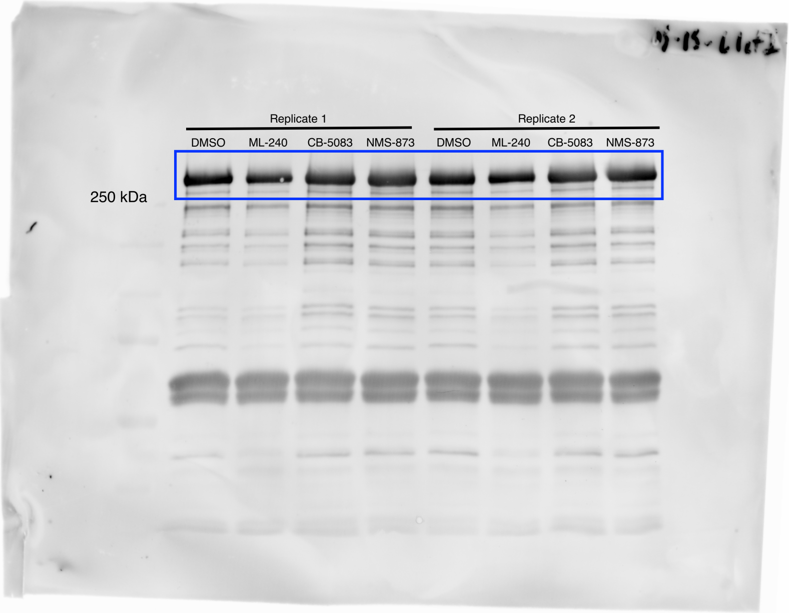
Replicate 1
Replicate 2
DMSO
ML-240
CB-5083
NMS-873
DMSO
ML-240
CB-5083
NMS-873
250 kDa
